# Combined Effects of Metals and PFAS Exposure on Prevalent Diabetes

**DOI:** 10.3390/jox16040128

**Published:** 2026-07-10

**Authors:** Rifa Tasnia, Emmanuel Obeng-Gyasi

**Affiliations:** 1 Department of Built Environment, North Carolina A&T State University, Greensboro, NC 27411, USA; 2 Environmental Health and Disease Laboratory, North Carolina A&T State University, Greensboro, NC 27411, USA

**Keywords:** environmental chemical mixtures, diabetes prevalence, Bayesian Kernel Machine Regression (BKMR), weighted quantile sum (WQS) regression, quantile g-computation, PFAS (per- and polyfluoroalkyl substances), heavy metals, exposure mixture analysis

## Abstract

Human per- and polyfluoroalkyl substances (PFAS) and metal exposures occur as mixtures, but most studies evaluate them independently. Using data from adults in the 2017–2018 National Health and Nutrition Examination Survey (NHANES) with directly measured PFAS and blood metals (N = 1648), we assessed joint associations of perfluorooctanoic acid (PFOA), perfluorooctanesulfonic acid (PFOS), lead, cadmium, and mercury with prevalent diabetes via survey-weighted logistic regression, Weighted Quantile Sum (WQS) regression, quantile g-computation, and design-aware and naïve Bayesian Kernel Machine Regression (BKMR). Survey-weighted diabetes prevalence was 11.4%. Lead and PFOS emerged as the dominant contributors to the exposure mixture in the BKMR model, with posterior inclusion probabilities of 1.00 and 0.997, respectively. In the WQS model, PFOS and PFOA were the primary drivers of the positive mixture index, consistent with their proposed roles in endocrine disruption and insulin resistance. BKMR exposure-response functions were non-monotonic, indicating that lead and cadmium associations with diabetes are nonlinear rather than uniformly directional across the exposure range, a structure that conventional logistic regression cannot capture. Overall, restricting to directly measured exposures, incorporating the full NHANES design in the primary regression, and triangulating across complementary mixture frameworks provide a more rigorous platform than prior single-pollutant or design-naive approaches.

## 1. Introduction

About 589 million people worldwide suffer from diabetes mellitus, a serious public health issue that is expected to affect over 850 million people by 2050 [1]. It is a long-term metabolic condition characterized by elevated blood sugar levels. The emphasis has recently shifted away from established risk factors due to growing evidence that environmental exposures, particularly hazardous chemicals, may also contribute to the start and progression of diabetes. For example, elevated blood lead levels (BLL) have been linked to several risk factors for diabetes, and lead is known to upset the hormonal balance [2].

Along with well-known lifestyle factors like food and exercise, environmental pollution is becoming more widely acknowledged as a possible risk factor for diabetes [3,4]. Emerging evidence in epidemiology suggests that exposure to heavy metals like lead and cadmium may increase the risk of type 2 diabetes [5]. In addition to being significant risk factors for high blood glucose, environmental contaminants may work in concert to increase metabolic risk [6]. A wide range of studies has reported links between diabetes and exposures to air pollution, heavy metals, persistent organic pollutants (POPs), PFAS, and phthalates [7,8]. These environmental chemicals may contribute to diabetes by affecting insulin sensitivity, damaging pancreatic β-cells, and causing oxidative stress [6]. Although people are typically exposed to multiple substances at once, most of this study has focused on individual exposures [9]. In practice, people are exposed to multiple environmental chemicals simultaneously, and the health effects of these exposures may differ when they occur together rather than individually [10].

Despite declining trends in serum concentrations of legacy PFAS over two decades of national biomonitoring, more than 96% of the U.S. general population retains measurable concentrations of PFOS and PFOA, including individuals born after manufacturing phase-outs began in 2000–2002, indicating persistent widespread exposure [11,12].

Numerous studies have identified a significant association between exposure to heavy metals, particularly lead (Pb) and cadmium (Cd), and prevalent diabetes, largely due to their detrimental effects on pancreatic function [10,13]. Multiple metal exposures have been associated with prevalent diabetes in prior research, although the direction and magnitude of these relationships may vary by metal and population [10]. Lead may interfere with glucose metabolism by promoting hepatic gluconeogenesis and reducing pancreatic beta-cell activity, according to experimental studies. This may lead to the development of type 2 diabetes and glucose intolerance [14,15,16]. Lead is a common environmental contaminant that has been shown to have detrimental effects on human health [17]. Due to recent industrialization, lead has become a common heavy metal contributing to widespread environmental contamination in China [2].

A large cross-sectional study of U.S. adults using NHANES 1999–2020 data demonstrated that serum cadmium was significantly associated with diabetes risk, with the direction and magnitude of associations varying across exposure levels—consistent with a potential nonlinear dose–response relationship [18,19].

Diabetes risk models have traditionally relied on anthropometric measurements (BMI, waist circumference, blood pressure), laboratory biomarkers (insulin, lipids, liver enzymes), behavioral factors (smoking, alcohol consumption), and demographic factors (age, sex, race/ethnicity, socioeconomic status) [20,21]. There is an ongoing debate over the connection between diabetes and heavy metals. Heavy metals and diabetes were found to be positively correlated in several studies, whereas other investigations reported no correlation or a negative correlation [10,22,23,24,25,26]. In addition, several epidemiological studies have investigated the relationship between heavy metal exposure and broader metabolic disorders, including metabolic syndrome, although the reported associations have remained inconsistent across different populations [23]. Previous studies have reported altered metal concentrations among individuals with diabetes, suggesting that metal-related biological processes may be involved in glucose metabolism and metabolic dysfunction [27].

Principal component analysis (PCA), multiple linear regression (MLR), and cluster analysis are examples of traditional analytical techniques. They frequently have limited capacity to describe complex nonlinear and interactive effects within environmental exposure mixtures, especially when interactions or nonlinear correlations among exposures are involved. More sophisticated techniques, such as Bayesian Kernel Machine Regression (BKMR) and quantile g-computation (qgcomp), have been developed to overcome this. For evaluating the combined and interaction effects of multiple environmental exposures on metabolic outcomes, sophisticated mixture modeling techniques such as BKMR and WQS regression are useful [6]. These techniques allow researchers to investigate the complex and mixed effects of different exposures in more detail [28,29,30,31].

BKMR is an adaptable technique that can examine the association between multiple exposures and an outcome without requiring rigid assumptions about the relationship’s structure. It is especially useful for identifying interactions and nonlinear effects among different exposures while adjusting for other factors [32,33]. In mixture analysis, additional techniques such as quantile g-computation and weighted quantile sum (WQS) regression are useful because they identify the most influential contributors and estimate the overall impact of combined exposures [30]. As diabetes is increasing and people are widely exposed to harmful environmental chemicals, it is important to understand how these chemicals work together to affect health.

PFAS compounds and toxic metals may influence diabetes through overlapping biological pathways, including oxidative stress, inflammation, endocrine disruption, impaired glucose homeostasis, and pancreatic beta-cell dysfunction. Therefore, evaluating these exposures as mixtures may provide additional insight beyond individual pollutant analyses. Prior studies of PFAS and diabetes have reported mixed findings, including positive, null, and inverse associations, and causal interpretation remains uncertain because many studies are observational or cross-sectional.

A recent synthesis of cross-sectional and prospective evidence found inverse associations between long-chain PFAS, particularly PFDA, and type 2 diabetes risk in older adults, and identified a nonlinear dose–response relationship between PFOA exposure and diabetes risk [12]. These heterogeneous findings underscore the importance of evaluating PFAS–diabetes relationships within a mixture framework capable of accommodating nonlinear and context-dependent exposure effects [12].

Although associations between individual metals and PFAS compounds and diabetes have been reported previously, humans are exposed to these contaminants as complex mixtures rather than as isolated chemicals. Evaluating exposures one chemical at a time may therefore underestimate the health effects associated with concurrent environmental exposures. Furthermore, studies evaluating the combined effects of metals and PFAS within a unified mixture-modeling framework remain limited, and it remains unclear which exposures contribute most consistently to the overall mixture signal across different analytical approaches [2,34].

Previous NHANES mixture studies often included participants who did not have PFAS measurements. Instead, they estimated (imputed) PFAS levels for these participants, which can introduce exposure misclassification bias and affect the accuracy of exposure–outcome associations [11]. To our knowledge, no previous study has limited the analysis to participants with directly measured PFAS and metal biomarkers while also accounting for the complex NHANES design.

To address these gaps, this study examined the combined associations of two PFAS (PFOA and PFOS) and three blood metals (lead, cadmium, and mercury) with self-reported diabetes among U.S. adults using NHANES 2017–2018 data. We included only participants with directly measured PFAS and metal biomarker concentrations, avoiding the bias that can occur when PFAS values are imputed for participants without measurements [11].

Unlike previous studies that focused mainly on single exposures or did not fully account for the NHANES design, we used survey-weighted logistic regression along with three mixture methods—Bayesian Kernel Machine Regression (BKMR), Weighted Quantile Sum (WQS) regression, and quantile g-computation. These methods were used to evaluate the combined effects of the exposures, identify the most important contributors to the mixture, and compare results across different analytical approaches. The primary survey-weighted logistic regression incorporated PFAS subsample sampling weights (WTSB2YR), primary sampling units (SDMVPSU), and strata (SDMVSTRA) to support design-based inference, whereas the WQS, quantile g-computation, and BKMR mixture analyses were conducted as sample-level secondary analyses because current implementations do not natively accommodate the complex survey design.

### Objectives

**Figure 1:**
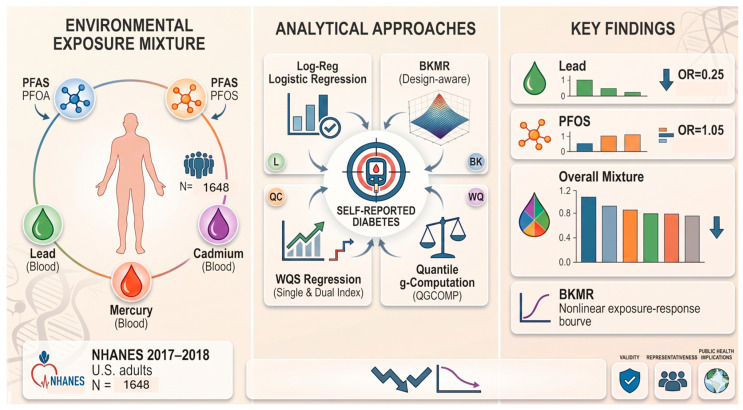
An analytical workflow that uses quantile g-computation, WQS regression, and BKMR to assess the relationship between prevalent diabetes and environmental chemical mixtures.

The primary objective of this study is to investigate the joint associations between environmental chemical mixtures and prevalent diabetes among U.S. adults, incorporating the complex NHANES design to enable population-representative inference (Figure 1).

The specific objectives are:

1. To use survey-weighted logistic regression, limited to individuals with directly measured biospecimen concentrations for all five exposures, to assess the combined and individual associations of blood metals (lead, cadmium, and mercury) and PFAS compounds (PFOA and PFOS) with prevalent self-reported diabetes.
2. To use BKMR to characterize nonlinear, concentration-specific exposure-response relationships between individual environmental exposures and diabetes risk, paying special attention to effects that occur within the low-to-moderate exposure range.
3. To assess the relative importance of individual exposures within the mixture using posterior inclusion probabilities (PIPs) from BKMR, exposure-specific weights from dual-index WQS regression, and directional contributions from quantile g-computation, and also to evaluate the consistency of variable importance findings across complementary analytical frameworks.
4. To examine the overall joint mixture effect on diabetes prevalence using WQS regression and quantile g-computation, and to evaluate the robustness of findings through pre-specified sensitivity analyses including eGFR adjustment, lab-confirmed diabetes outcome definition, complete-case analysis, and exclusion of high-exposure outliers.

## 2. Materials and Methods

### 2.1. Data Source and Study Population

The National Health and Nutrition Examination Survey (NHANES) 2017–2018, conducted by the Centers for Disease Control and Prevention (CDC) through the National Center for Health Statistics (NCHS), provided the publicly available data used in this investigation. NHANES employs a stratified, multistage probability sampling design to produce nationally representative estimates of the U.S. civilian non-institutionalized population [35].

The analytic sample for this study was restricted to adult participants (aged ≥ 18 years) who had both PFAS and blood metals directly measured in NHANES 2017–2018. Because PFAS biomarkers are assessed only in a random one-third subsample of examined participants, this restriction was essential to ensure that all exposure variables were based on direct biospecimen measurement rather than imputation across subsamples. Participants with missing values on the primary outcome (self-reported diabetes) were additionally excluded. The resulting measured-only analytic sample comprised 1648 participants. This sample restriction addressed a critical methodological concern: the prior analysis had imputed PFAS concentrations for participants outside the PFAS subsample, a practice that introduces non-ignorable bias in exposure-outcome associations. Exposure variables were not imputed under any circumstances; only covariate values were subject to imputation, as described in Section 2.5.

### 2.2. Outcome Definition

Diabetes status was ascertained using the NHANES Diabetes Questionnaire (DIQ010), which asked participants whether they had ever been informed by a doctor or other health professional that they have diabetes or sugar diabetes, excluding gestational diabetes where applicable. Participants who responded “Yes” were classified as having diabetes (coded 1), and those who responded “No” were classified as not having diabetes (coded 0). Participants who responded “borderline,” “refused,” “don’t know,” or had missing responses were excluded from the analysis.

Self-reported physician-diagnosed diabetes was selected as the primary outcome because it provides a precisely defined binary measure suitable for logistic and mixture modeling frameworks while maintaining adequate analytic sample size. As a sensitivity analysis, a lab-confirmed diabetes definition was also evaluated, operationalized as HbA1c ≥ 6.5% or self-reported use of diabetes medication. Results from both outcome definitions are reported.

### 2.3. Exposure Assessment

Environmental exposures included five biomarkers measured directly in NHANES 2017–2018 biospecimens:

- PFAS compounds: Perfluorooctanoic acid (PFOA) and perfluorooctanesulfonic acid (PFOS), measured in serum (ng/mL)
- Blood metals: Lead, cadmium, and mercury, measured in whole blood (µg/dL)

These five exposures were selected based on prior evidence linking them individually and in combination to metabolic dysfunction, insulin resistance, and diabetes risk [2,5,6,13]. All five biomarkers were directly measured for each participant in the analytic sample; no exposure imputation was performed. Because all five exposures were right-skewed, each was natural-log transformed and then standardized (mean = 0, standard deviation = 1) prior to analysis to improve model stability and allow comparability of effect estimates across exposures with different units and distributions. Accordingly, odds ratios for the exposures correspond to a one-standard-deviation increase in log-transformed concentration.

### 2.4. Covariates

Potential confounders were selected based on prior epidemiological evidence and biological plausibility. The following covariates were included in all models:

- Demographic: age (continuous, years), sex (male/female), race/ethnicity (Non-Hispanic White, Mexican American, Other Hispanic, Non-Hispanic Black, Non-Hispanic Asian, Other/Multiracial)
- Socioeconomic: educational attainment (less than high school, high school graduate, some college, college graduate, above college), income-to-poverty ratio (continuous)
- Behavioral: smoking status (current, former, never), alcohol use (current drinker, past year drinker, none past year)
- Clinical: body mass index (BMI, continuous, kg/m^2^)

### 2.5. Survey Design and Sampling Weights

To obtain population-representative estimates and appropriately account for the complex NHANES probability sampling design, all primary analyses incorporated the PFAS subsample weights (WTSB2YR), which are defined for all participants with PFAS measurements and adjust for unequal selection probabilities, nonresponse, and poststratification. The primary sampling unit variable (SDMVPSU) and stratum variable (SDMVSTRA) were included to account for clustering and stratification, respectively. Because the analytic sample was restricted to participants with directly measured PFAS, WTSB2YR was defined for all included participants, enabling design-aware analyses without further weight adjustment.

Survey-weighted logistic regression was implemented using the svyglm function from the survey package in R, specifying a quasibinomial family with logit link. This approach properly propagates design uncertainty into standard errors and confidence intervals. The design-aware BKMR framework followed the weight-proportional PSU resampling approach described by Jehu-Appiah and Obeng-Gyasi [36], which approximates a weighted empirical distribution by resampling observations within strata proportional to their survey weights while preserving the primary sampling unit structure.

### 2.6. Missing Data and Imputation

Following restriction of the analytic sample to participants with directly measured PFAS and blood metals (N = 1698 with measured exposures; N = 1648 with non-missing diabetes outcome), covariate missingness was substantially lower than in the full NHANES adult sample. Specifically, smoking status was complete (0% missing), alcohol use was missing for 6.7% of participants, and education level was missing for 5.2% of participants, compared to the 37–45% missingness reported in the prior unrestricted analysis. This reduction confirms that the elevated missingness previously observed was attributable to merging participants across subsamples with different data collection protocols rather than true item nonresponse.

Covariates with residual missingness were handled using multiple imputation by chained equations (MICE) [37,38], with predictive mean matching for continuous variables and polytomous logistic regression for categorical variables. The outcome variable and all exposure variables were not imputed. Five imputed datasets were generated (m = 5) with 10 iterations each (maxit = 10). A complete-case sensitivity analysis (N = 1274) was conducted to assess the robustness of results to the imputation approach. Estimates were consistent between the imputed and complete-case analyses, as reported in the Supplementary Material.

### 2.7. Statistical Analysis

#### 2.7.1. Descriptive Statistics and Correlation Analysis

Participant characteristics were summarized using means and standard deviations for continuous variables, and frequencies and percentages for categorical variables. Survey-weighted diabetes prevalence was estimated using svymean with the PFAS subsample design. Spearman rank correlation coefficients (ρ) were computed among the five exposure variables to characterize pairwise collinearity.

#### 2.7.2. Survey-Weighted Logistic Regression

Survey-weighted multivariable logistic regression was used to estimate the independent association of each exposure with self-reported diabetes, adjusting for all covariates listed in Section 2.4. The model was specified as:

$$logit[P(Y=1)]=\beta_{0}+\beta_{1}Z_{1}+\ldots +\beta_{5}Z_{5}+\gamma X$$

where *Y* denotes diabetes status, *Z*_1_–*Z*_5_ denote standardized exposure variables, and *X* denotes the covariate vector. All analyses used the svyglm function with PFAS subsample weights (WTSB2YR), SDMVPSU, and SDMVSTRA. Odds ratios (ORs) and 95% confidence intervals (CIs) were computed. A formal Lead × PFOA interaction term was additionally tested to evaluate whether the association of lead with diabetes varied across levels of PFOA exposure, as motivated by shared mechanisms of metabolic disruption [6,13].

#### 2.7.3. Bayesian Kernel Machine Regression (BKMR)

BKMR was applied to flexibly estimate the joint, nonlinear, and potentially interactive effects of the five-exposure mixture on diabetes risk [32]. The model is specified as:

g[E(Y|Z, X)]=h(Z)+Xγ

where *h*(*Z*) is an unknown smooth exposure–response function estimated using a Gaussian kernel, *Z* = (*Z*_1_, …, *Z*_5_) is the vector of standardized exposures, *X* is the covariate matrix, and *g*(·) is the logit link. Variable selection was enabled (varsel = TRUE) and PIPs were used to summarize the relative importance of each exposure. PIPs close to 1 indicate strong evidence that the exposure contributes to the exposure–response surface.

Both a naïve (unweighted) BKMR and a design-aware BKMR were implemented, following the PSU-level weight-proportional resampling framework of this study [36]. In the design-aware approach, PSUs were resampled with replacement within strata proportional to their mean survey weight, and individuals were resampled within selected PSUs proportional to their individual weights, producing a weighted empirical distribution as input to standard BKMR fitting. Both models were run for 20,000 MCMC iterations across four independent chains. Convergence was assessed using the Gelman–Rubin potential scale reduction factor ($\hat{R}$) [39] and visual inspection of trace plots. PIP stability was evaluated by comparing PIPs across chains.

Mixture effects were summarized through: (1) posterior inclusion probabilities, (2) univariate exposure–response functions (holding all other exposures at their median), (3) single-variable risk summaries (change in response from 25th to 75th percentile of each exposure), and (4) overall mixture effect (joint change in response when all exposures shift from their 25th to 75th percentiles).

#### 2.7.4. Weighted Quantile Sum (WQS) Regression

WQS regression was applied to estimate the overall directional effect of the exposure mixture on diabetes risk while addressing multicollinearity among correlated exposures [40]. Exposure variables were transformed into quartiles and combined into a weighted index:

$$WQS=\Sigma \;{ŵ}_{j}\;q_{j},\;\mathrm{where}\;\Sigma \;{ŵ}_{j}=1\;\mathrm{and}\;{ŵ}_{j}\geq 0$$

where ŵ_j_ is the estimated weight for the *j*-th exposure and *q_j_* is its quartile-transformed value. A dual-index WQS approach was implemented to separately estimate positive (pwqs) and negative (nwqs) mixture indices, allowing for bidirectional effects within the same model [41]. Bootstrap resampling (B = 100) with a 40% training/60% test split was used to estimate stable weights. Note that WQS was implemented without survey weights, as current gWQS implementations do not natively support complex sampling designs; results are therefore interpreted as sample-level associations and reported as a secondary analysis.

#### 2.7.5. Quantile g-Computation (qgcomp)

Quantile g-computation was applied to estimate the joint effect of simultaneously increasing all five exposures by one quartile [30]. The model is specified as:

$$logit[P(Y=1)]=\psi_{0}+\psi_{1}\Sigma q_{j}+X\gamma$$

where *ψ*_1_ = *Σβ_j_* is the overall mixture effect obtained by summing the exposure-specific regression coefficients, and *X* is the covariate vector. Unlike WQS, quantile g-computation allows exposures to contribute in both positive and negative directions simultaneously. Exponentiation of *ψ*_1_ yields an odds ratio representing the change in diabetes odds associated with a one-quartile simultaneous increase in all exposures. Both a no-bootstrap estimate (delta-method confidence intervals) and a bootstrapped estimate (B = 200) were obtained. Quantile g-computation was applied without survey weights and results are interpreted as sample-level associations.

#### 2.7.6. Sensitivity Analyses

Three pre-specified sensitivity analyses were conducted. First, a complete-case analysis (N = 1274) was performed to assess robustness to covariate imputation. Second, eGFR was estimated from serum creatinine (Chronic Kidney Disease Epidemiology Collaboration [CKD-EPI] equation) and added as an additional covariate in the survey-weighted logistic model to assess whether renal function confounds the metal–diabetes associations. Third, the primary logistic regression model was repeated using a lab-confirmed diabetes outcome (HbA1c ≥ 6.5% or self-reported diabetes medication use) to evaluate outcome misclassification.

### 2.8. Statistical Software

All analyses were conducted in R version 4.3 (R Core Team, Vienna, Austria). Survey-weighted analyses used the survey package. BKMR was implemented using the bkmr package [32]. WQS regression used gWQS [30,42]. Quantile g-computation used qgcomp [30]. Multiple imputation used mice [43]. Data manipulation and visualization used dplyr, tidyr, and ggplot2. Convergence diagnostics used coda. All code is available upon request.

### 2.9. Analytical Strategy Summary

This revised analysis addressed three key methodological limitations of the prior submission. First, the analytic sample was restricted to participants with directly measured PFAS and blood metals (N = 1648), eliminating imputed exposure values for participants outside the PFAS subsample. Second, the primary survey-weighted logistic regression incorporated the NHANES complex survey design through PFAS subsample weights, PSU clustering, and stratification to enable design-based inference, while the mixture analyses (WQS, quantile g-computation, and BKMR) were retained as sample-level secondary analyses. Third, BKMR was extended to four chains with 20,000 iterations each, with formal convergence diagnostics, and a design-aware BKMR version was implemented alongside the naïve model to evaluate the influence of survey design on mixture inference. Together, these revisions substantially strengthen the methodological rigor and interpretability of the reported associations.

## 3. Results

### 3.1. Study Population

**Figure 2:**
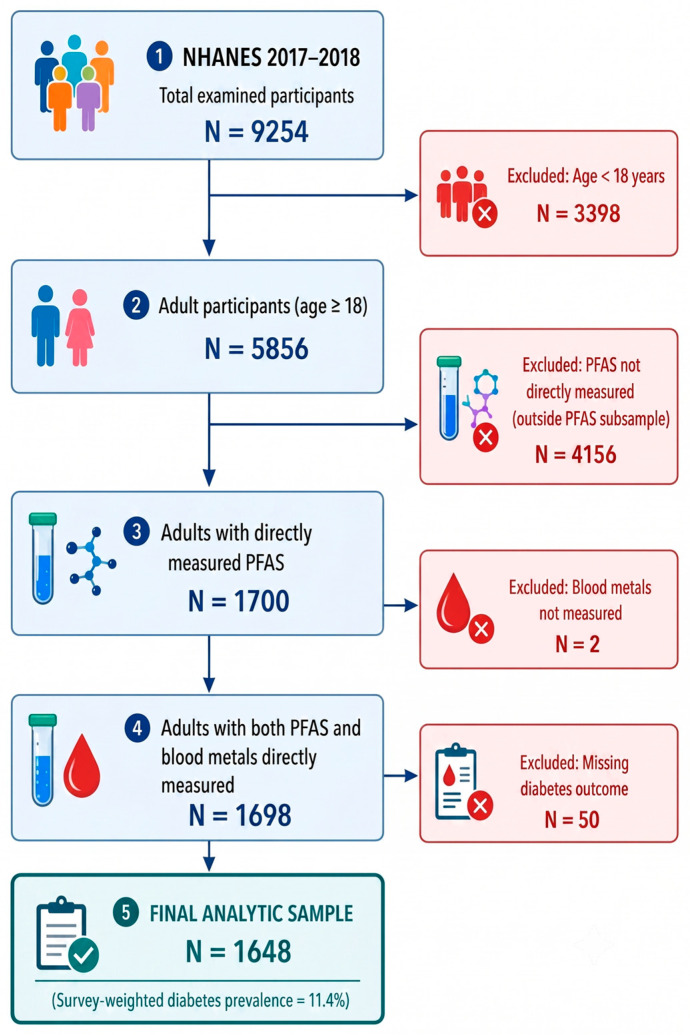
Derivation of the analytic sample (NHANES 2017–2018).

The measured-only analytic sample consisted of 1648 adults from NHANES 2017–2018 with directly measured serum PFAS and blood metal concentrations. The survey-weighted prevalence of self-reported diabetes was 11.4% (SE = 1.09%), whereas 250 participants (15.2%) reported physician-diagnosed diabetes in the unweighted sample. Participant selection is illustrated in Figure 2.

Table 1 summarizes the sociodemographic and clinical characteristics of the analytic sample. Of the 1648 participants, 49.3% were male and 50.7% were female, with a mean age of 49.2 ± 18.7 years. Non-Hispanic White participants constituted the largest racial/ethnic group (35.9%), followed by Non-Hispanic Black (21.9%), Mexican American (14.5%), Non-Hispanic Asian (13.1%), Other Hispanic (9.5%), and Other/Multiracial participants (5.2%). Approximately one-third of participants were college graduates (31.7%), while 22.0% had education beyond the college level. Most participants had never smoked (60.2%) and reported alcohol consumption during the previous year (65.6%). The mean body mass index was 29.7 ± 7.8 kg/m^2^, and the mean income-to-poverty ratio was 2.50 ± 1.61, indicating substantial demographic, socioeconomic, and behavioral diversity within the study population.

### 3.2. Exposure Characteristics

#### 3.2.1. Distribution of Environmental Exposures

The distributions of the five environmental exposure biomarkers are summarized in Table 2.

Table 2 summarizes the descriptive statistics of the five environmental exposure biomarkers included in the present study. PFOS exhibited the highest median concentration (4.70 ng/mL) and the greatest overall variability (mean ± SD: 6.87 ± 7.75 ng/mL), whereas cadmium showed the lowest median concentration (0.31 µg/L). Blood lead (median: 0.90 µg/dL), mercury (median: 0.68 µg/L), and PFOA (median: 1.50 ng/mL) demonstrated intermediate concentration ranges with substantial inter-individual variability. The relatively large standard deviations compared with the corresponding median values indicate considerable heterogeneity in exposure levels across participants.

**Figure 3:**
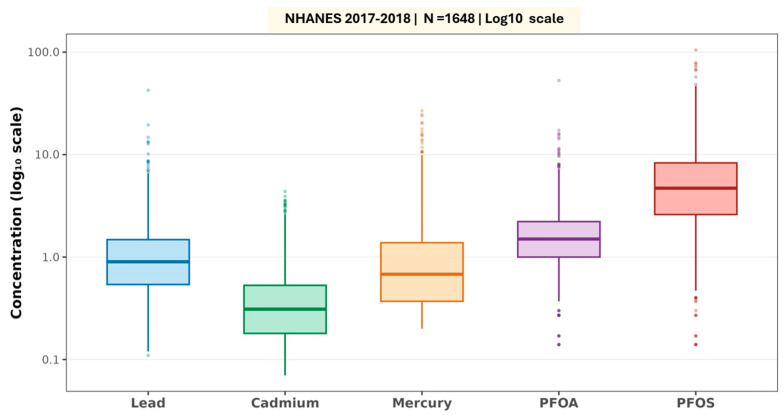
Distribution of environmental exposure biomarkers among participants in the measured-only analytic sample (NHANES 2017–2018, N = 1648).

To further visualize the distributional characteristics of these biomarkers, boxplots are presented in Figure 3.

Figure 3 illustrates that all five environmental contaminants exhibited positively skewed distributions with numerous observations in the upper tail, a pattern commonly observed in human biomonitoring studies. PFOS demonstrated the highest median concentration and greatest variability, whereas cadmium exhibited the lowest median concentration. The substantial heterogeneity and presence of extreme values suggest that conventional linear models may not adequately characterize exposure–response relationships. These distributional characteristics therefore support the application of flexible mixture-modeling approaches, including BKMR, WQS regression, and quantile g-computation, to evaluate nonlinear and joint exposure effects.

Boxes represent the interquartile range (IQR), center lines indicate medians, whiskers extend to 1.5 × IQR, and individual points denote observations beyond the whiskers. Concentrations are displayed on a log10 scale to facilitate comparison across biomarkers with different concentration ranges. Blood metals (lead, cadmium, and mercury) were measured in whole blood, whereas PFAS (PFOA and PFOS) were measured in serum.

#### 3.2.2. Correlation Structure of Environmental Exposures

**Figure 4:**
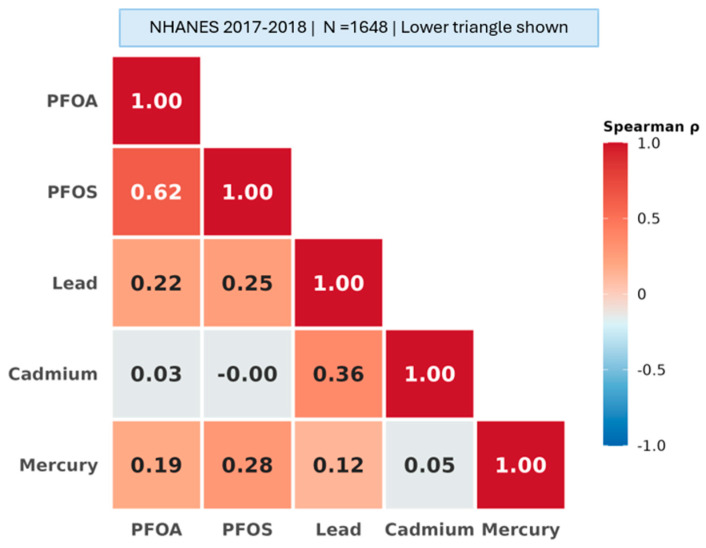
Spearman correlation matrix of serum PFAS and blood metal biomarkers in the measured-only analytic sample (NHANES 2017–2018, N = 1648). Cells display Spearman’s rank correlation coefficients (ρ), with color intensity representing the strength and direction of correlation. Positive correlations are shown in red and negative correlations in blue. Statistical significance is indicated as *p* < 0.05, *p* < 0.01, and *p* < 0.001.

Spearman rank correlation coefficients among the five environmental exposures are shown in Figure 4. Overall, pairwise correlations ranged from −0.00 to 0.63, showing low-to-moderate correlations among the exposures. The strongest positive correlation was found between PFOA and PFOS (ρ = 0.63, *p* < 0.001), which matches their shared sources of environmental exposure. Moderate positive correlations were also found between lead and cadmium (ρ = 0.36, *p* < 0.001), PFOS and mercury (ρ = 0.28, *p* < 0.001), PFOS and lead (ρ = 0.25, *p* < 0.001), and PFOA and lead (ρ = 0.22, *p* < 0.001). Weaker but still statistically significant positive correlations were seen between PFOA and mercury (ρ = 0.19, *p* < 0.001), lead and mercury (ρ = 0.12, *p* < 0.001), and cadmium and mercury (ρ = 0.05, *p* < 0.05). In contrast, there was almost no correlation between PFOS and cadmium (ρ = −0.00) or between PFOA and cadmium (ρ = 0.03).

Even though no pairwise correlation went above the usual threshold for severe multicollinearity (ρ ≥ 0.70), the correlation structure suggests that these environmental contaminants are not fully independent. Therefore, statistical methods that can model correlated exposures together, such as BKMR, WQS regression, and quantile g-computation, were used to analyze the combined effects of the environmental mixture.

These findings indicate moderate correlation among several contaminants while remaining below conventional thresholds for severe multicollinearity. Consequently, mixture-modeling approaches capable of jointly evaluating correlated exposures were applied in subsequent analyses.

Pairwise correlations among the environmental exposures were generally weak to moderate, indicating no evidence of severe multicollinearity. Additional multicollinearity diagnostics are presented in Supplementary Table S3.

#### 3.2.3. Unadjusted Diabetes Prevalence Across Exposure Quartiles

**Figure 5:**
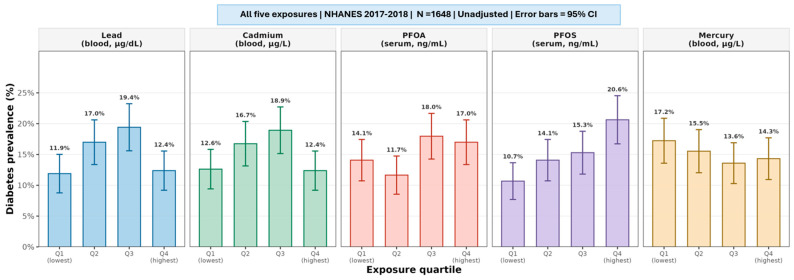
Unweighted prevalence of self-reported diabetes across quartiles of all five environmental exposures (NHANES 2017–2018, N = 1648). Each panel presents the proportion of participants reporting physician-diagnosed diabetes within each exposure quartile (Q1 = lowest, Q4 = highest concentration). Error bars represent 95% confidence intervals based on the Wald method. Blood metals (lead, cadmium, mercury) were measured in whole blood; PFAS compounds (PFOA, PFOS) were measured in serum. Estimates are unadjusted and do not reflect survey-weighted or covariate-adjusted associations. Non-monotonic patterns for lead and cadmium—with prevalence peaking in Q3 before declining in Q4—are consistent with the nonlinear exposure–response relationships identified in subsequent BKMR analyses.

Figure 5 presents the unweighted prevalence of self-reported diabetes across quartiles of all five environmental exposures, with exact concentration ranges for each quartile provided to facilitate interpretation alongside the BKMR exposure–response functions (Section 3.4.4).

For blood lead, diabetes prevalence showed a striking non-monotonic pattern. Prevalence was 11.9% in Q1 (0.11–0.54 µg/dL), rose to 17.0% in Q2 (0.54–0.90 µg/dL) and 19.4% in Q3 (0.90–1.48 µg/dL), then declined to 12.4% in Q4 (1.48–42.48 µg/dL). Q1 through Q3—spanning 0.11 to 1.48 µg/dL—represent the range where approximately 75% of participants are found, and within this range, diabetes prevalence rose progressively by 7.5 percentage points. The apparent decline at Q4 encompasses an extremely wide concentration range (1.48 to 42.48 µg/dL), indicating considerable heterogeneity within this quartile. These crude estimates are consistent with the nonlinear, non-monotonic BKMR exposure–response function for lead (Section 3.4.4).

For blood cadmium, a closely analogous non-monotonic pattern was observed. Prevalence rose from 12.6% in Q1 (0.07–0.18 µg/L) to 16.7% in Q2 (0.18–0.31 µg/L) and 18.9% in Q3 (0.31–0.53 µg/L), then declined to 12.4% in Q4 (0.53–4.37 µg/L). Within Q1–Q3, diabetes prevalence rose by 6.3 percentage points across concentrations experienced by the large majority of participants.

For serum PFOS, prevalence increased monotonically from 10.7% in Q1 (0.14–2.60 ng/mL) to 20.6% in Q4 (8.30–104.90 ng/mL)—a total increase of 9.9 percentage points. This monotonic crude pattern is notable given that PFOS showed a non-significant logistic regression OR (1.05, *p* = 0.631) after covariate adjustment, suggesting that the unadjusted association is confounded by other variables, and that the BKMR-identified contribution (PIP = 0.997) reflects nonlinear and co-exposure-dependent effects not captured by either crude or adjusted regression estimates.

For serum PFOA, a non-monotonic pattern was observed (Q1: 14.1%, Q2: 11.7%, Q3: 18.0%, Q4: 17.0%) with no consistent directional trend. For blood mercury, prevalence showed a slight inverse pattern (Q1: 17.2%, Q2: 15.5%, Q3: 13.6%, Q4: 14.3%), consistent with the near-null associations in logistic regression and BKMR.

These unadjusted patterns are consistent with but do not confirm the nonlinear exposure-response relationships characterized in Section 3.4.

### 3.3. Survey-Weighted Logistic Regression Analyses

#### 3.3.1. Primary Survey-Weighted Logistic Regression

Survey-weighted multivariable logistic regression models were fitted to evaluate the independent associations between each environmental exposure and prevalent self-reported diabetes after adjustment for demographic, socioeconomic, behavioral, and clinical covariates.

Table 3 presents the adjusted associations between environmental exposure biomarkers and prevalent self-reported diabetes after controlling for demographic, socioeconomic, behavioral, and clinical covariates. Blood lead demonstrated the strongest inverse association with diabetes (OR = 0.25, 95% CI: 0.09–0.71, *p* = 0.020), followed by blood cadmium (OR = 0.71, 95% CI: 0.54–0.94, *p* = 0.028). Serum PFOA exhibited a non-significant inverse trend (OR = 0.66, 95% CI: 0.42–1.04, *p* = 0.092), whereas PFOS and mercury were not significantly associated with diabetes. Among the adjustment covariates, increasing age and higher BMI were independently associated with greater odds of diabetes. Compared with Non-Hispanic White participants, Non-Hispanic Asian participants had significantly higher odds of diabetes, whereas Other Hispanic participants had significantly lower odds. Higher educational attainment was also independently associated with reduced odds of diabetes.

**Figure 6:**
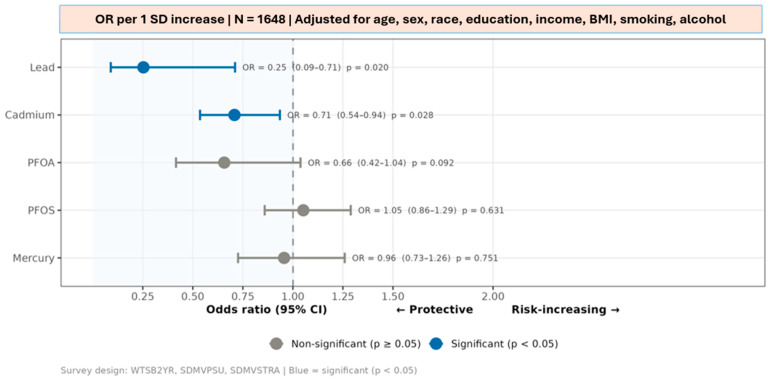
Survey-weighted multivariable logistic regression estimates for the associations between PFAS and metal exposures and prevalent self-reported diabetes among U.S. adults (NHANES 2017–2018, N = 1648). Odds ratios (ORs) and 95% confidence intervals (CIs) are shown for a one-standard-deviation increase in each log-transformed, standardized exposure. The dashed vertical line indicates the null association (OR = 1).

Figure 6 provides a visual summary of the relative magnitude, direction, and precision of the adjusted associations between each environmental exposure and prevalent diabetes. Compared with the numerical estimates presented in Table 3, the forest plot facilitates direct comparison of effect sizes and confidence intervals across all exposures. Blood lead exhibited the largest inverse association with diabetes, followed by blood cadmium. Serum PFOA demonstrated a modest inverse trend; however, its confidence interval crossed the null value. In contrast, PFOS and mercury produced effect estimates close to unity, indicating little evidence of an independent association with diabetes. Collectively, these findings indicate that the inverse associations observed in the fully adjusted models were primarily driven by blood lead and cadmium and provide the reference framework for interpreting the subsequent interaction, nonlinear, and mixture analyses.

Results from complete-case analysis are presented in Supplementary Table S1.

#### 3.3.2. Lead × PFOA Interaction Analysis

**Figure 7:**
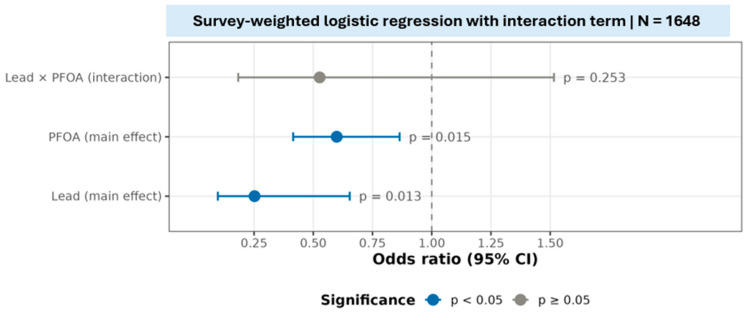
Lead × PFOA interaction analysis from the survey-weighted logistic regression model. Odds ratios (ORs) and 95% confidence intervals (CIs) are shown for the main effects of lead and PFOA and their interaction term. The dashed vertical line represents the null value (OR = 1).

As shown in Figure 7, the survey-weighted interaction model did not provide evidence of a statistically significant interaction between lead and PFOA exposures in relation to diabetes prevalence (interaction *p* = 0.253). This null interaction result indicates that the present study provides little empirical support for biologically meaningful synergistic interactions between lead and PFOA at current population-level exposure concentrations.

The estimated interaction effect was centered near the null value and its confidence interval crossed unity, suggesting no detectable departure from additivity on the multiplicative scale. However, the main effects of both lead (*p* = 0.013) and PFOA (*p* = 0.015) remained statistically significant in the interaction model, indicating that both exposures were independently associated with diabetes prevalence when evaluated simultaneously.

#### 3.3.3. Nonlinear Dose–Response Relationships

**Figure 8:**
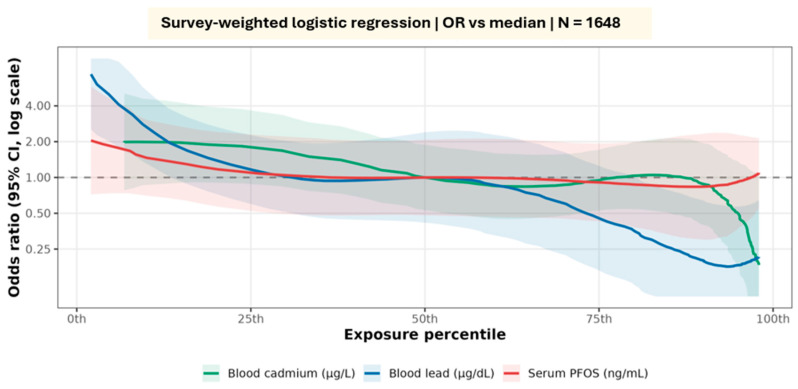
Restricted cubic spline (RCS) dose–response relationships between selected environmental exposures and prevalent self-reported diabetes estimated using survey-weighted logistic regression models (NHANES 2017–2018, N = 1648). Odds ratios (ORs) are presented relative to the median (50th percentile) exposure level for blood lead (µg/dL), blood cadmium (µg/L), and serum PFOS (ng/mL). Natural cubic splines were fitted using knots at the 25th, 50th, and 75th percentiles of each exposure distribution. Shaded bands represent 95% confidence intervals. The dashed horizontal line indicates the null association (OR = 1), and the *y*-axis is displayed on a logarithmic scale. Survey design was accounted for using PFAS subsample weights (WTSB2YR), primary sampling units (SDMVPSU), and strata (SDMVSTRA). PFOS: perfluorooctanesulfonic acid.

To further characterize potential nonlinear exposure–response relationships, restricted cubic spline (RCS) models were fitted within the survey-weighted logistic regression framework using knots placed at the 25th, 50th, and 75th percentiles of each exposure distribution, with the median (50th percentile) serving as the reference value (Figure 8). Blood lead demonstrated a pronounced inverse nonlinear association with prevalent diabetes.

The estimated odds ratio declined steeply across the lower exposure percentiles before gradually approaching a plateau at higher concentrations, suggesting that the inverse association was strongest within the lower-to-middle exposure range. Blood cadmium also exhibited evidence of nonlinearity, with elevated odds observed at lower concentrations, a nadir near the median exposure level, and a gradual decline at higher concentrations, although confidence intervals widened substantially at the upper end of the exposure distribution. In contrast, serum PFOS displayed a relatively flat exposure–response curve centered around the null value across most of the exposure range, consistent with the absence of a statistically significant association in the primary survey-weighted logistic regression model. Overall, these findings indicate that nonlinear modeling provides additional insight beyond conventional linear regression and broadly supports the exposure–response patterns identified using BKMR.

Overall, the primary findings remained consistent across multiple complementary analyses, including interaction analyses, restricted cubic spline modeling, subgroup analyses stratified by sex and race/ethnicity, and sensitivity analyses excluding extreme exposure values (Supplementary Figures S3, S4, and S7). These analyses collectively support the robustness of the observed inverse associations for blood lead and cadmium.

### 3.4. Bayesian Kernel Machine Regression (BKMR) Analysis

#### 3.4.1. Convergence Diagnostics

Model convergence was assessed using visual inspection of MCMC trace plots together with the Gelman–Rubin potential scale reduction factor ($\hat{R}$). Detailed convergence diagnostics are presented in Supplementary Figures S1, S5 and S6 and Supplementary Table S3.

The naïve BKMR model converged, with the four chains in close agreement (Gelman–Rubin $\hat{R}$ = 1.03–1.08, below the conventional 1.10 threshold). The survey-weighted (design-aware) BKMR did not converge: despite four independent chains of 20,000 iterations, the Gelman–Rubin potential scale reduction factors ranged from 3.05 to 13.16 (Supplementary Table S3), far exceeding the 1.10 threshold, and the trace plots showed persistent separation among chains (Supplementary Figure S1). Because posterior summaries such as posterior inclusion probabilities and exposure–response functions are not estimable from chains that have not converged, primary BKMR inference was based on the converged naïve model; the design-aware results are reported only for methodological transparency and are not interpreted. The primary epidemiologic conclusions were corroborated using the survey-weighted logistic regression analyses.

#### 3.4.2. Posterior Inclusion Probabilities (PIPs)

**Figure 9:**
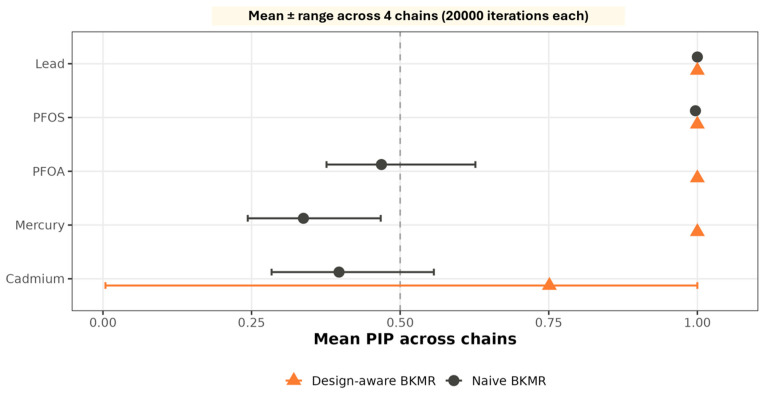
PIPs from naïve and design-aware BKMR models (NHANES 2017–2018, N = 1648). Points represent mean PIPs across four independent MCMC chains of 20,000 iterations each; horizontal bars indicate the observed range across chains. The dashed vertical line at PIP = 0.50 indicates the conventional variable inclusion threshold. Naïve BKMR (gray circles) converged (Gelman–Rubin $\hat{R}$: 1.03–1.08) and is used for primary inference. Design-aware BKMR (orange triangles) did not converge ($\hat{R}$: 3–13) and is shown for methodological transparency only; it is not interpreted.

Posterior inclusion probabilities (PIPs) from both the naïve and survey-weighted BKMR models, estimated across four independent MCMC chains of 20,000 iterations each, are presented in Figure 9. PIP stability across the four chains for the naïve BKMR model is shown in Supplementary Figure S5.

In the naïve BKMR model, lead exhibited a mean PIP of 1.000 across all four chains with negligible range, indicating near-certain and highly stable inclusion in the exposure–response function. PFOS demonstrated a mean PIP of 0.997 with a similarly narrow range, also providing strong evidence of inclusion. A high PIP reflects statistical importance within the exposure-response surface as modeled by BKMR; that is, inclusion of PFOS improves the model’s characterization of the joint exposure-response relationship and should not be interpreted as evidence of an independent biological effect or causal role for PFOS in diabetes etiology. Given the substantial PFOA-PFOS correlation (ρ = 0.63), the high PIP for PFOS may partly reflect its association with correlated co-exposures within the modeled mixture. In contrast, PFOA (mean PIP = 0.469), mercury (mean PIP = 0.337), and cadmium (mean PIP = 0.397) fell below the conventional 0.50 threshold, suggesting comparatively weaker independent contributions after accounting for within-mixture correlation. Gelman–Rubin R-hat statistics ranged from 1.03 to 1.08 for the naïve model, confirming adequate chain convergence.

The design-aware BKMR model produced substantially different PIP estimates with considerably wider ranges across chains—most notably, cadmium showed a mean PIP near 0.75 but with a range spanning nearly the full 0–1 interval. Gelman–Rubin $\hat{R}$ statistics for the design-aware model ranged from 3 to 13, far above the 1.10 threshold, indicating that its chains did not converge; these estimates are therefore not interpreted.

Numerical comparison with WQS variable weights is presented in Supplementary Table S2.

#### 3.4.3. Overall Mixture Effect

**Figure 10:**
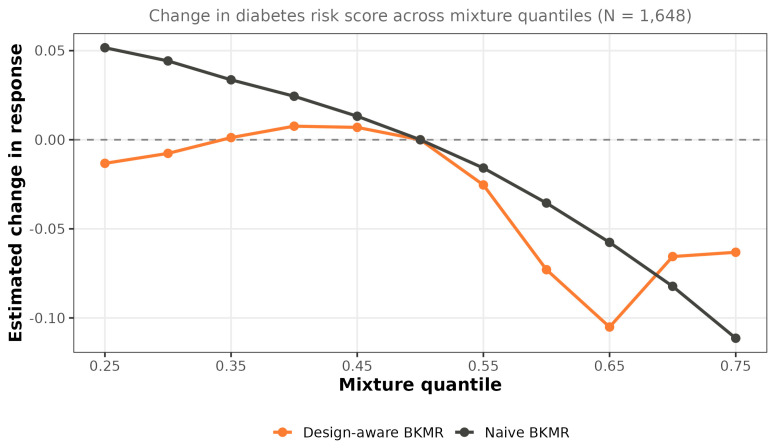
Estimated overall mixture effects from the BKMR models across exposure mixture quantiles. The reference level was fixed at the 50th percentile of the mixture distribution. The horizontal dashed line indicates no change in the estimated response relative to the reference level (change in response = 0).

The estimated overall mixture effect is presented in Figure 10. This figure shows the change in estimated diabetes risk as the combined exposure mixture shifts from lower to higher quantiles, with the 50th percentile serving as the reference.

The overall mixture-response curve was non-monotonic and should be interpreted across the exposure distribution rather than from a single summary. Across the 0.25–0.75 mixture quantile range, where most participants are concentrated, the estimated response varied with the joint exposure level rather than following a strictly linear trend, with lead and cadmium—the components with the highest posterior inclusion probabilities—likely the principal contributors to this shape. This non-monotonic structure is the feature that conventional regression and linear-in-quantile mixture summaries average over; it is not, by itself, evidence of an overall harmful or overall inverse joint association. The overall direction of the joint association is more directly estimated by quantile g-computation (Section 3.5), which indicates an inverse association.

Beyond the 0.75 mixture quantile, where relatively few participants were observed, the estimated response declines toward and below the reference level. This apparent reversal should not be interpreted as evidence that very high mixture exposure is associated with lower diabetes risk, as estimates in this range are based on relatively sparse data and should be interpreted cautiously.

Instead, it most likely reflects the limited number of observations at the extreme upper tail of the exposure distribution, resulting in greater estimation uncertainty. Consequently, the response estimates in this region should be interpreted cautiously. Unlike logistic regression, which summarizes an average association across the entire exposure distribution, BKMR reveals how the estimated effect changes across different exposure levels. When interpreted together with the univariate exposure-response functions (Section 3.4.4), these results indicate that the estimated joint association varies across the exposure distribution, underscoring that a single average effect can obscure non-monotonic structure; the net direction of the joint association across this range was inverse in the directly comparable quantile g-computation analysis (Section 3.5).

#### 3.4.4. Univariate Exposure-Response Functions

**Figure 11:**
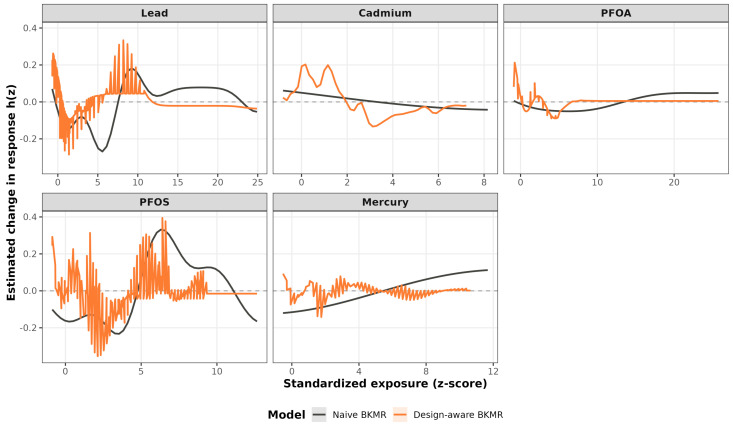
Univariate exposure–response functions estimated from naïve and survey design-aware BKMR models. Curves represent the estimated change in diabetes response across the observed exposure range while holding all other exposures at their median levels. The horizontal dashed lines indicate no change in the estimated response (*h*(z) = 0).

The univariate exposure–response functions estimated from the naïve BKMR model are presented in Figure 11. These curves illustrate how the estimated diabetes response changes across the observed exposure distribution for each chemical while holding all remaining exposures at their median values. Unlike conventional logistic regression, which estimates a single average association across the entire exposure distribution, BKMR allows the exposure–response relationship to vary with exposure level and therefore provides a more detailed characterization of nonlinear effects.

Lead. Lead demonstrated the strongest nonlinear exposure–response relationship. Between approximately z = −1 and z = +2, corresponding to blood lead concentrations of approximately 0.2–1.5 μg/dL, where the majority of study participants were concentrated, increasing lead exposure was associated with an increase in the estimated diabetes response, though this pattern should be interpreted with caution given BKMR estimation uncertainty at moderate sample sizes. This represents the most epidemiologically relevant portion of the curve because it reflects the exposure range experienced by most individuals in the study population. Beyond approximately z = +2, corresponding to the extreme upper tail of the exposure distribution where relatively few participants were observed, the estimated response gradually flattened and then declined. Therefore, the inverse odds ratio observed in the survey-weighted logistic regression represents an average association across the entire exposure distribution and does not fully capture the exposure-range-specific pattern identified by BKMR.

Cadmium. Cadmium showed a similar nonlinear relationship. Across approximately z = 0 to z = +3 (approximately 0–1.5 μg/L), where most participants were observed, increasing cadmium exposure was associated with an increase in the estimated diabetes response. At higher exposure levels, where relatively few observations were available, the curve became relatively stable. These findings are consistent with potential variation in the estimated association between cadmium and diabetes across the exposure distribution rather than remaining constant.

PFOS. PFOS exhibited a nonlinear exposure–response pattern. Between approximately z = 0 and z = +5, the estimated diabetes response generally increased with increasing exposure before stabilizing at higher concentrations. Although PFOS was not significantly associated with diabetes in the survey-weighted logistic regression, it had one of the highest posterior inclusion probabilities (PIP = 0.997), indicating a statistical contribution to the overall mixture-response surface that was not detectable by marginal regression—though this should not be interpreted as evidence of an independent biological or causal role for PFOS.

PFOA. PFOA demonstrated relatively small changes across most of its observed exposure range. A slight increase in the estimated response was observed at lower exposure levels, after which the curve remained relatively flat. These findings are consistent with its intermediate posterior inclusion probability (PIP = 0.469) and the absence of a statistically significant association in the survey-weighted logistic regression.

Mercury. Mercury showed little evidence of a substantial nonlinear exposure–response relationship. The estimated response remained close to the reference value throughout most of the observed exposure distribution, consistent with its comparatively low posterior inclusion probability (PIP = 0.337) and the near-null association observed in the survey-weighted logistic regression.

Overall, the BKMR exposure–response functions demonstrate that the estimated effects of individual environmental chemicals vary across the exposure distribution. Importantly, the greatest changes in the estimated diabetes response occurred within the low-to-moderate exposure ranges where most participants were concentrated. These nonlinear patterns illustrate the principal advantage of BKMR, which identifies the exposure ranges where the mixture may have its greatest impact, whereas conventional logistic regression provides only a single average association across the entire exposure distribution.

#### 3.4.5. Single-Variable Risk Summaries

**Figure 12:**
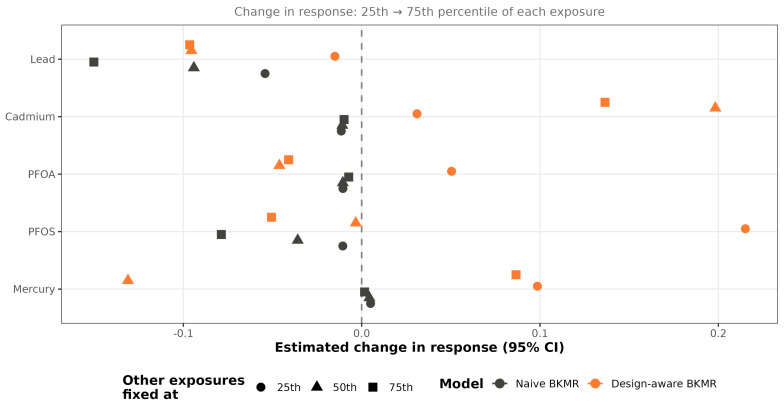
Estimated change in diabetes response associated with increasing each exposure from the 25th to the 75th percentile while holding all remaining exposures fixed at the 25th, 50th, or 75th percentile. The vertical dashed line at zero indicates no estimated change in response.

Figure 12 presents the single-variable risk summaries, showing the estimated change in diabetes risk when each exposure is increased from its 25th to 75th percentile while holding the remaining exposures fixed at their 25th, 50th, or 75th percentile. These summaries provide an overall estimate of the contribution of each individual exposure within the context of the entire chemical mixture.

Lead showed consistently negative summary estimates across all three background exposure levels. However, these summary estimates should be interpreted together with the univariate exposure-response functions (Figure 11). The single-variable summary represents an average effect across the exposure distribution, whereas the univariate BKMR curves demonstrate that the estimated effect of lead varies across exposure levels. In particular, the BKMR response curves indicate that the estimated effect of lead varies non-monotonically across the exposure range rather than remaining constant. Thus, the single-variable summary alone does not fully capture the nonlinear exposure-response relationship identified by BKMR.

Cadmium exhibited greater variability across background exposure levels than lead, with a positive estimated response when the remaining exposures were fixed at higher background concentrations and near-null or slightly negative responses at lower background levels. This finding suggests that the estimated effect of cadmium may vary depending on the overall mixture context. PFOS, PFOA, and mercury showed comparatively smaller and less consistent single-variable effects across background exposure levels, consistent with their lower posterior inclusion probabilities relative to lead.

These findings further illustrate the value of BKMR for mixture analysis. Unlike conventional regression models, which estimate a single average association for each exposure, BKMR demonstrates that the estimated effect of an individual chemical depends on both its exposure level and the background levels of the remaining mixture components. Consequently, interpretation of environmental mixtures requires consideration of the entire exposure-response surface rather than a single summary estimate.

#### 3.4.6. Assessment of Exposure Interactions

**Figure 13:**
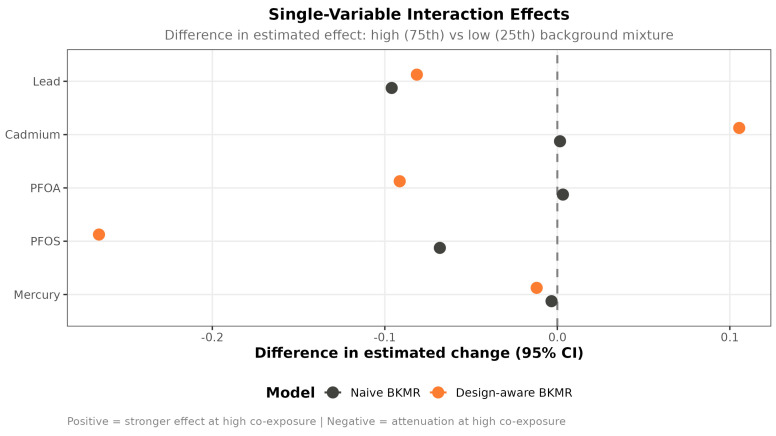
Differences in estimated exposure effects under high (75th percentile) versus low (25th percentile) background mixture levels obtained from BKMR models. The vertical dashed line at zero indicates no difference in the estimated exposure effect between the high and low background mixture levels.

Figure 13 summarizes the single-variable interaction effects by comparing the estimated change in response when the background mixture is fixed at the 75th percentile versus the 25th percentile. Positive values indicate a stronger estimated effect at higher background mixture levels, whereas negative values indicate attenuation of the estimated effect under higher co-exposure conditions.

In the naïve BKMR model, which is used for primary inference, the single-variable interaction estimates were generally close to zero, indicating minimal variation in exposure effects across background mixture levels. The corresponding design-aware estimates are displayed in Figure 13 for methodological transparency only and are not interpreted, because that model did not converge (Gelman–Rubin $\hat{R}$ = 3–13).

The corresponding interaction estimates from the naïve BKMR model were generally close to zero, indicating minimal variation in exposure effects across different background mixture levels. Overall, these results suggest that any potential interaction effects within the environmental mixture were relatively modest and exposure-specific.

### 3.5. Weighted Quantile Sum Regression and Quantile G-Computation

#### Weighted Quantile Sum Regression

**Figure 14:**
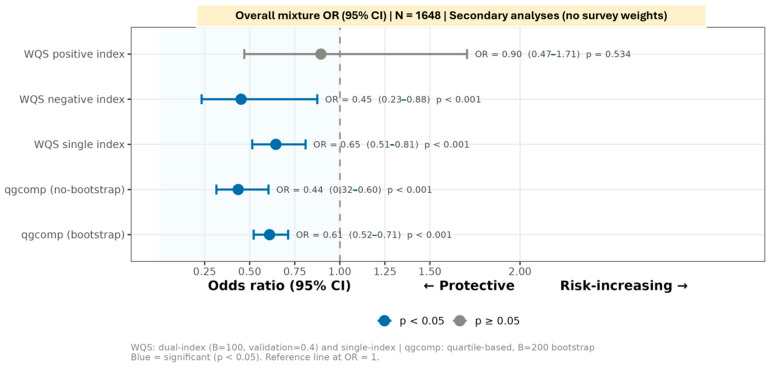
Overall mixture effect estimates from WQS regression and qgcomp. ORs and 95% CIs are shown for the dual-index WQS positive and negative mixture indices, the single-index WQS model, and qgcomp models with and without bootstrap resampling. The vertical dashed line indicates the null value (OR = 1). Blue points indicate statistically significant associations (*p* < 0.05), whereas gray points indicate non-significant associations (*p* ≥ 0.05).

WQS regression was conducted as a secondary sample-level analysis without survey weights, as current gWQS implementations do not natively support complex survey designs; results should be interpreted as sample-level associations. Results are presented in Table 4 and Figure 14.

The overall mixture effect estimates obtained from WQS regression and qgcomp are presented in Figure 14. Both methods consistently indicated an overall inverse association between the environmental mixture and prevalent self-reported diabetes.

The positive mixture index in the dual-index WQS model was not statistically significant (OR = 0.90, 95% CI: 0.47–1.71, *p* = 0.534), whereas the negative mixture index was significantly associated with lower odds of diabetes (OR = 0.45, 95% CI: 0.23–0.88, *p* < 0.001). Similarly, there was a significant inverse association (OR = 0.65, 95% CI: 0.51–0.81, *p* < 0.001) with the traditional single-index WQS model.

Quantile g-computation produced highly consistent findings. Significant inverse associations were observed using both the non-bootstrap model (OR = 0.44, 95% CI: 0.31–0.60, *p* < 0.001) and the bootstrap implementation (OR = 0.61, 95% CI: 0.52–0.71, *p* < 0.001), demonstrating that the estimated mixture effect was robust across alternative statistical approaches.

Collectively, Figure 14 shows a high degree of agreement between WQS regression and qgcomp, with all models consistently identifying an inverse overall association between the environmental mixture and prevalent diabetes. The concordance across these complementary mixture-based approaches strengthens confidence in the observed mixture effect and provides independent support for the primary findings. Exposure-specific WQS weights are summarized in Supplementary Table S2.

**Figure 15:**
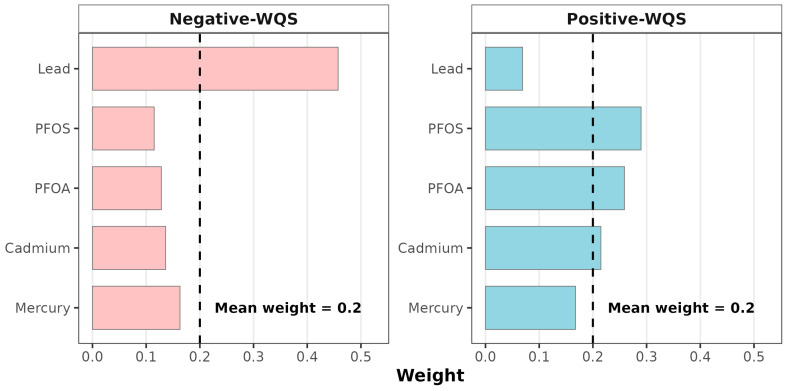
The dual-index WQS regression model yielded estimated component weights. The contributions of each exposure to the positive and negative mixture indices are displayed in separate panels. The expected weight under equal contribution (0.20) is shown by the dashed vertical line. Exposures with weights greater than 0.20 make a stronger-than-anticipated contribution to the relevant mixture index.

Figure 15 presents the exposure-specific weights estimated from the dual-index WQS regression model, illustrating the relative contribution of each contaminant to the positive and negative mixture indices.

Blood lead was the main contributor to the negative index, making up 45.8% of the mixed weight (weight = 0.458), which was much higher than the equal-weight criterion of 0.20. PFOA (0.128), PFOS (0.096), cadmium (0.136), and mercury (0.163) all made somewhat lesser contributions to the inverse mixture component.

Cadmium (0.187), mercury (0.168), and lead (0.064) contributed relatively little to the positive mixture component, while PFOS (weight = 0.354) and PFOA (weight = 0.231) were the main drivers of the positive index, both of which surpassed the equal-weight criterion.

These findings indicate that different contaminants contributed disproportionately to the two mixture indices, with blood lead predominantly characterizing the inverse association, while PFOS and PFOA were the principal contributors to the positive index. The distinct weighting patterns further suggest that the overall mixture effect was driven by different exposure profiles depending on the direction of the association.

### 3.6. Results of Sensitivity Analyses 

#### 3.6.1. eGFR-Adjusted and Lab-Confirmed Diabetes Models

**Figure 16:**
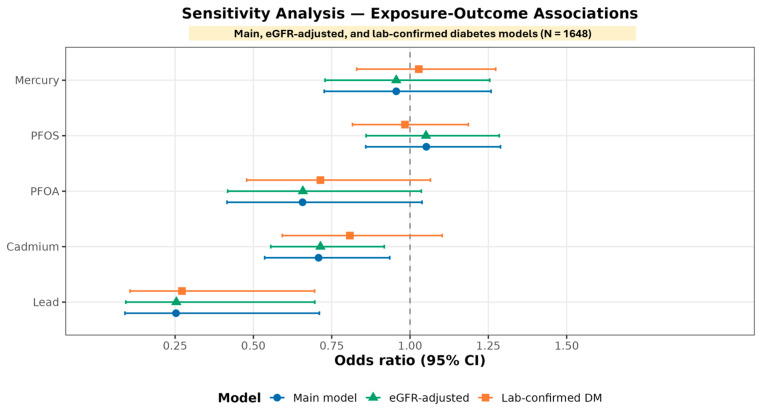
Sensitivity analyses that contrast eGFR-adjusted and laboratory-confirmed diabetes models with the main survey-weighted logistic regression model. For every exposure, odds ratios (ORs) and 95% confidence intervals (CIs) are shown. The null value (OR = 1) is shown by the dashed vertical line.

To evaluate the robustness of the primary findings, two complementary sensitivity analyses were performed. First, the primary survey-weighted logistic regression model was additionally adjusted for estimated glomerular filtration rate (eGFR), calculated from serum creatinine using the Chronic Kidney Disease Epidemiology Collaboration (CKD-EPI) equation, to assess potential confounding by renal function. Second, alternate outcome criteria based on either self-reported use of diabetes medication or laboratory-confirmed diabetes (HbA1c ≥ 6.5%) were used to repeat the study. Figure 16 displays the findings from these analyses.

The primary findings remained largely unchanged across both sensitivity analyses. Blood lead continued to demonstrate a significant inverse association with diabetes in both the eGFR-adjusted model (OR ≈ 0.25, *p* = 0.018) and also the laboratory-confirmed diabetes model (OR ≈ 0.27, *p* = 0.016). Blood cadmium likewise remained inversely associated with diabetes under both alternative modeling strategies. In contrast, serum PFOA, PFOS, and mercury remained non-significantly associated with diabetes across both sensitivity analyses. The consistency of these findings indicates that the observed inverse associations for blood lead and cadmium were robust to adjustment for kidney function and to the use of an alternative diabetes outcome definition, supporting the stability of the primary results.

#### 3.6.2. Complete-Case Sensitivity Analysis

**Figure 17:**
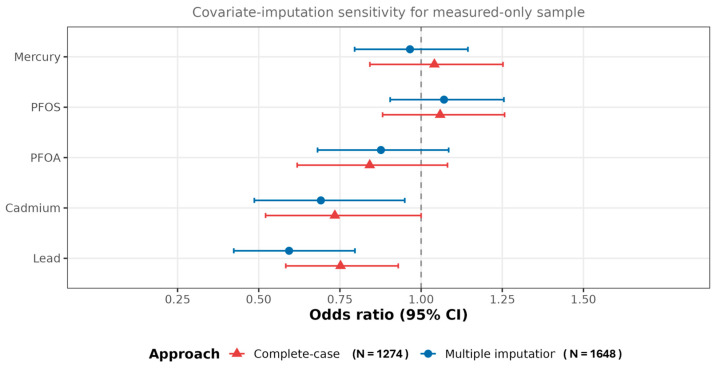
Comparison of complete-case and multiple-imputation analyses for the measured-only sample. Odds ratios (ORs) and 95% confidence intervals (CIs) are shown for each exposure under both analytical approaches. The vertical dashed line at OR = 1 indicates the null value.

Because missing covariate data may influence effect estimates, we compared results from complete-case analysis (N = 1274) with multiple imputation findings (Figure 17). The estimated associations were highly consistent between approaches, with lead and cadmium remaining inversely associated with diabetes under both analytic strategies. The similarity of effect estimates and confidence intervals supports the robustness of the primary findings to missing data assumptions.

## 4. Discussion

The principal contribution of the present study is methodological rather than biological: the restriction to directly measured exposures, the incorporation of the NHANES complex survey design, and the application of complementary mixture modeling frameworks provide a more rigorous and transparent platform than prior studies have employed.

In this nationally representative analysis of U.S. adults from NHANES 2017–2018, we evaluated the joint associations of two per- and polyfluoroalkyl substances (PFOA and PFOS) and three metals (lead, cadmium, and mercury) with prevalent diabetes using survey-weighted logistic regression together with multiple complementary mixture modeling approaches. Restricting the analysis to participants with direct measurements of all environmental exposures minimized exposure misclassification while preserving the complex design of the NHANES [44].

The principal contribution of the present study is methodological rather than biological: the restriction to directly measured exposures, the incorporation of the NHANES complex survey design into all primary analyses, and the application of complementary mixture modeling frameworks provide a more rigorous and transparent analytical platform for evaluating PFAS-metal mixture associations with diabetes than prior studies have employed.

In survey-weighted logistic regression, blood lead and cadmium were inversely associated with prevalent diabetes, while PFOS, mercury, and PFOA were not significantly associated. BKMR identified lead and PFOS as the most statistically influential contributors to the exposure-response surface, with lead exhibiting a posterior inclusion probability of 1.000 and PFOS of 0.997. The dual-index WQS model identified lead as the dominant contributor to the negative mixture index, while PFOS and PFOA dominated the positive index. WQS regression and quantile g-computation consistently estimated an inverse overall mixture association. These findings are broadly consistent with prior epidemiological literature identifying lead as a dominant predictor in metal-PFAS mixture analyses, and the multiple analytical frameworks strengthen confidence in the robustness of this conclusion through methodological triangulation [32,45].

### 4.1. Interpretation of the Lead and Cadmium Associations

Lead and cadmium’s inverse relationships in the survey-weighted logistic regression should not be taken as proof that these metals prevent diabetes. Both lead and cadmium cause oxidative stress, mitochondrial dysfunction, pancreatic β-cell damage, chronic inflammation, and impaired glucose metabolism, all of which are biologically associated with an elevated risk of diabetes, according to numerous experimental and epidemiological studies [2,6,46,47,48].

Numerous methodological issues could account for the apparent inverse associations found in this study. First, because NHANES is cross-sectional, it is impossible to determine temporal relationships because blood metal concentrations and diabetes status are measured simultaneously. Second, people with diabetes usually alter their diet, work, and lifestyle, which may lower their exposure to the environment in the future. This could lead to reverse causation. Third, the clearance kinetics of several environmental pollutants may be altered by kidney dysfunction, resulting in intricate correlations between circulating concentrations and disease status. Lastly, survivor bias and residual confounding may potentially be responsible for inverse relationships in observational studies.

Crucially, the BKMR analyses offer more information than the logistic regression estimates. The naïve-BKMR univariate functions were non-monotonic, with the largest changes occurring within the densely populated low-to-moderate exposure range and attenuation confined largely to sparsely populated high-exposure regions. These patterns are hypothesis-generating and do not establish a net harmful joint effect; rather, they indicate that biologically relevant nonlinear structure within the population-relevant exposure range may be obscured by logistic regression odds ratios, which reflect average effects across the entire exposure distribution.

The increase in estimated diabetes risk observed across the Q1-to-Q3 concentration range (11.9% to 19.4% for lead) is consistent with a potential nonlinear exposure-response pattern; however, given the moderate sample size (N = 1648) and the inherent estimation uncertainty in BKMR exposure-response functions, this interpretation should remain cautious. These crude quartile patterns are unadjusted and may reflect confounding, and the BKMR curves, while supportive, do not provide definitive evidence of nonlinearity.

### 4.2. Importance of Nonlinear Mixture Modeling

Environmental contaminants rarely occur in isolation, and individuals are typically exposed to multiple correlated chemicals simultaneously through shared environmental and dietary sources [45]. Conventional regression models estimate the independent association of each exposure while generally assuming linearity and additivity, assumptions that may not adequately represent real-world environmental mixtures.

The correlation analysis demonstrated that several environmental contaminants were moderately correlated, particularly PFOA with PFOS and lead with cadmium, reflecting shared environmental sources and exposure pathways. Although the observed correlations were insufficient to indicate problematic multicollinearity, they suggest that the exposures do not occur independently in the general population. This exposure correlation structure supports the application of mixture-based analytical approaches, such as BKMR, WQS regression, and quantile g-computation, which can simultaneously evaluate correlated exposures while accounting for their potentially nonlinear and joint effects [30,32,40]. Such approaches provide a more realistic representation of real-world environmental exposures than conventional single-pollutant regression models [30,32].

In addition to the primary regression analyses, restricted cubic spline models suggested that the associations between environmental exposures and diabetes were not uniformly linear. Blood lead demonstrated a pronounced inverse nonlinear exposure–response relationship, whereas blood cadmium exhibited evidence of a non-monotonic association with greater uncertainty at higher exposure concentrations. In contrast, serum PFOS remained centered around the null across most of the observed exposure range. These findings are broadly consistent with the BKMR analyses, which also identified nonlinear exposure–response functions, and suggest that flexible modeling approaches may better capture the complexity of environmental exposure–disease relationships than conventional linear models alone. Similar nonlinear relationships have been reported previously for several environmental contaminants, highlighting the importance of considering nonlinearity when evaluating chronic low-level environmental exposures.

BKMR addresses these limitations by simultaneously modeling nonlinear exposure-response relationships and interactions among mixture components. In the present study, BKMR identified lead and PFOS as the dominant contributors to the overall mixture despite the absence of a statistically significant association for PFOS in the survey-weighted logistic regression. This discrepancy illustrates that an exposure with little independent marginal association may nevertheless play an important role within the overall exposure mixture when considered jointly with correlated co-exposures.

The formal Lead × PFOA interaction test provided no evidence of multiplicative effect modification (*p* = 0.253), and the present study therefore provides little empirical support for biologically meaningful synergistic interactions among these contaminants at current population-level exposure concentrations. The biological rationale presented in Section 4.6 should accordingly be interpreted as identifying plausible pathways consistent with the observed associations rather than as a tested mechanistic hypothesis. Statistical importance within the BKMR mixture model and biological relevance are related but not synonymous; the high posterior inclusion probability for PFOS reflects its statistical contribution to explaining the joint exposure-response surface and should not be interpreted as evidence of a causal or independent biological role.

Nonlinear dose–response relationships for environmental contaminants are increasingly documented in environmental epidemiology; the present findings extend this observation to the context of a PFAS-metal mixture in a nationally representative sample, though the exact functional form of these relationships remains uncertain given the moderate sample size and inherent BKMR estimation uncertainty. The restricted cubic spline analyses provide supportive frequentist evidence for a nonlinear pattern but do not eliminate this uncertainty.

### 4.3. Complementary Findings Across BKMR, WQS, and Quantile G-Computation

BKMR, WQS regression, and quantile g-computation are based on different statistical assumptions and provide complementary, rather than identical, information about the mixture.

The single-index WQS model showed a significant inverse association for the overall chemical mixture, while the dual-index WQS model helped separate exposures that contributed in opposite directions. In this analysis, lead accounted for nearly half of the total negative mixture weight, whereas PFOS and PFOA together explained most of the positive component of the mixture.

Quantile g-computation, which summarizes the joint effect under a linear-in-quantile assumption, estimated a net inverse mixture association, whereas BKMR—imposing no such assumption—revealed a non-monotonic exposure–response surface. The two are therefore complementary rather than redundant, with the BKMR surface decomposing the structure that the quantile g-computation average summarizes as a single direction. Overall, the consistency of findings across these complementary analytical frameworks strengthens confidence through methodological triangulation. All methods ultimately identify the same dominant exposure and the same overall direction of association; the principal value of applying multiple mixture methods lies in robustness confirmation rather than in generating fundamentally different biological insights. The consistency of findings strengthens confidence that lead is the predominant exposure associated with diabetes within this mixture, but does not fundamentally alter this central conclusion.

The consistency of findings across BKMR, WQS regression, and quantile g-computation strengthens confidence in the robustness of the central finding—that lead is the predominant exposure associated with diabetes within this mixture—but does not fundamentally alter this conclusion or generate different biological insights beyond what single-exposure analyses have previously established.

### 4.4. Biological Plausibility

Several biologically plausible pathways may explain the observed associations, though the present cross-sectional study cannot test mechanistic hypotheses directly, and the findings do not establish that these pathways operate in the study population. Lead has been shown to induce oxidative stress, impair mitochondrial function, disrupt pancreatic β-cell activity, and interfere with insulin secretion [2,48]. Cadmium similarly promotes oxidative damage, alters zinc-dependent enzymatic pathways essential for insulin synthesis, and impairs glucose homeostasis [2,48]. PFAS compounds, particularly PFOS and PFOA, have been associated with endocrine disruption, altered lipid metabolism, chronic inflammation, activation of peroxisome proliferator-activated receptors, and insulin resistance.

Many environmental pollutants affect the body in similar ways [45]. When people are exposed to more than one pollutant, the combined effects may be stronger or different than exposure to a single pollutant. This makes the biological process more complex. Therefore, studying pollutant mixtures is important to better understand the environmental causes of diabetes.

### 4.5. Strengths

This study has several methodological strengths.

First, it included only participants with measured data for all environmental exposures, rather than estimating or filling in missing exposure values. This reduces measurement error and makes the exposure data more reliable. Second, all primary regression analyses used NHANES sampling weights, strata, and primary sampling units, which makes the results more representative of the U.S. adult population. Third, missing covariates were handled using multiple imputation, while exposure and outcome variables were kept unchanged. This approach helps reduce bias that can occur in complete-case analysis (where cases with missing data are simply removed). Fourth, multiple complementary mixture modeling approaches—including BKMR, WQS regression, and quantile g-computation—were applied, enabling evaluation of the consistency of findings across different statistical frameworks. Finally, several sensitivity analyses, including complete-case analyses, multiple-imputation analyses, and additional secondary analyses, demonstrated generally consistent findings.

Outlier sensitivity analysis confirmed that findings were not driven by extreme exposure values, and variance inflation factors confirmed the absence of multicollinearity among exposure variables.

The robustness of these findings is supported by multiple complementary analyses, including complete-case analysis, subgroup analyses, convergence diagnostics, and outlier sensitivity analyses (Supplementary Table S1; Supplementary Figures S3–S7). Collectively, these analyses demonstrated that the observed inverse associations for blood lead and cadmium remained directionally consistent across different analytical strategies and modeling assumptions, thereby strengthening confidence in the primary findings.

### 4.6. Limitations

This study has some limitations. First, because it is cross-sectional, we cannot say which comes first—environmental exposure or diabetes. Second, diabetes was mostly based on self-reports, but results were similar when using lab tests. Third, some unknown factors may still affect the results even after adjustment. Fourth, some advanced methods did not use survey weights, so they are only extra analyses, not the main results. Finally, the survey-weighted (design-aware) BKMR did not converge (Gelman–Rubin $\hat{R}$ = 3–13) despite four chains of 20,000 iterations; its results are shown alongside the naïve model for methodological transparency only and are not interpreted, and primary mixture inference relies on the converged naïve BKMR together with the survey-weighted logistic regression. Whether this survey-weighted mixture model can be made to converge in larger samples is left to future work.

## 5. Conclusions

This study shows that the relationship between diabetes and environmental chemical mixtures is complex, nonlinear, and not straightforward. Instead of evaluating single chemicals in isolation, the findings support assessing these exposures jointly rather than one chemical at a time. Among them, lead and PFOS were the biggest contributors to the overall exposure mixture. However, the results were not consistently simple—traditional analyses sometimes showed inverse associations between lead and cadmium (suggesting they were linked to lower diabetes risk, which is unexpected and may reflect complexity or confounding). The study also found that the effects vary with exposure level, meaning low, medium, and high exposure can show different patterns (nonlinear dose–response). Overall, different advanced mixture methods (BKMR, WQS regression, and quantile g-computation) yielded consistent results, supporting the idea that chemicals should be studied as mixtures rather than individually. Finally, the researchers suggest that future long-term cohort studies with repeated measurements and advanced statistical methods are needed to better understand causal links between environmental chemical mixtures and diabetes.

## Figures and Tables

**Table 1:** Distribution of sociodemographic and behavioral characteristics in the measured-only analytic sample (NHANES 2017–2018, N = 1648).

Variable	Category	*n* (%)	Category	*n* (%)
Sex	Male	813 (49.3)	Female	835 (50.7)
Age (years)	Mean ± SD	49.2 ± 18.7	Median (IQR)	50.0 (33.0–64.0)
Race/Ethnicity	Non-Hispanic White	591 (35.9)	Non-Hispanic Black	361 (21.9)
	Mexican American	239 (14.5)	Non-Hispanic Asian	216 (13.1)
	Other Hispanic	156 (9.5)	Other/Multiracial	85 (5.2)
Education	Less than high school	141 (8.6)	High school graduate	178 (10.8)
	Some college	358 (21.7)	College graduate	522 (31.7)
	Above college	362 (22.0)	Missing	87 (5.3)
Smoking status	Never	992 (60.2)	Former	369 (22.4)
	Current	287 (17.4)		
Alcohol use	Past year drinker	1081 (65.6)	None past year	299 (18.1)
	Never	160 (9.7)	Missing	108 (6.6)
BMI (kg/m^2^)	Mean ± SD	29.7 ± 7.8	Median (IQR)	28.3 (24.5–33.4)
Income-to-poverty ratio	Mean ± SD	2.50 ± 1.61	Median (IQR)	2.04 (1.17–4.00)

SD: standard deviation; IQR: interquartile range; BMI: body mass index. Income-to-poverty ratio missing for 218 participants (13.2%). All estimates are unweighted.

**Table 2:** Distribution of environmental exposures in the measured-only analytic sample (NHANES 2017–2018, N = 1648).

Exposure	Mean (SD)	Median	IQR	Unit
PFOA	1.89 (1.98)	1.50	1.00–2.27	ng/mL
PFOS	6.87 (7.75)	4.70	2.60–8.30	ng/mL
Lead	1.26 (1.65)	0.90	0.54–1.48	µg/dL
Cadmium	0.46 (0.48)	0.31	0.18–0.53	µg/L
Mercury	1.34 (2.18)	0.68	0.37–1.38	µg/L

Abbreviations: SD: standard deviation; IQR: interquartile range. PFOA: perfluorooctanoic acid; PFOS: perfluorooctanesulfonic acid. All exposures were right-skewed; distributions are presented on the original scale.

**Table 3:** Survey-weighted multivariable logistic regression examining associations between environmental exposures and self-reported diabetes among U.S. adults (NHANES 2017–2018, N = 1648).

Variable	OR	95% CI	*p*-Value
Exposure variables			
PFOA (per 1 SD)	0.66	0.42–1.04	0.092
PFOS (per 1 SD)	1.05	0.86–1.29	0.631
Lead (per 1 SD)	0.25	0.09–0.71	0.020
Cadmium (per 1 SD)	0.71	0.54–0.94	0.028
Mercury (per 1 SD)	0.96	0.73–1.26	0.751
Demographic variables			
Age (per year)	1.09	1.07–1.11	<0.001
Female (vs. male)	0.72	0.43–1.22	0.243
Race/Ethnicity (reference: Non-Hispanic White)			
Other Hispanic	0.51	0.28–0.92	0.041
Non-Hispanic Black	0.82	0.37–1.85	0.646
Non-Hispanic Asian	2.77	1.46–5.24	0.007
Other race	1.90	0.65–5.53	0.256
Education (reference: Less than high school)			
High school graduate	0.93	0.35–2.47	0.890
Some college	0.47	0.22–1.03	0.079
College graduate	0.46	0.27–0.78	0.011
Postgraduate or higher	0.34	0.15–0.77	0.020
Socioeconomic/Clinical variables			
Income-to-poverty ratio	1.08	0.92–1.27	0.358
BMI (per kg/m^2^)	1.07	1.03–1.11	0.005
Smoking (reference: Current smoker)			
Former smoker	0.89	0.34–2.30	0.811
Never smoker	0.46	0.14–1.48	0.210
Alcohol (reference: Current drinker)			
None during past year	1.55	0.77–3.14	0.241
Never drinker	0.93	0.37–2.34	0.877

Abbreviations: OR, odds ratio; CI, confidence interval; BMI, body mass index; SD, standard deviation. Survey-weighted multivariable logistic regression models incorporated NHANES PFAS subsample weights (WTSB2YR), primary sampling units (SDMVPSU), and stratification (SDMVSTRA). Odds ratios for environmental exposures represent a one-standard-deviation increase in log-transformed exposure concentrations.

**Table 4:** Mixture effect estimates from WQS regression and quantile g-computation (NHANES 2017–2018, N = 1648).

Model	Odds Ratio (OR)	95% Confidence Interval	*p*-Value
WQS positive index	0.90	0.47–1.71	0.534
WQS negative index	0.45	0.23–0.88	<0.001
WQS single index	0.65	0.51–0.81	<0.001
Quantile g-computation (without bootstrap)	0.44	0.31–0.60	<0.001
Quantile g-computation (bootstrap, B = 200)	0.61	0.52–0.71	<0.001

Abbreviations: OR, odds ratio; WQS, weighted quantile sum; qgcomp, quantile g-computation. WQS regression and quantile g-computation were conducted without survey weights as sample-level secondary analyses; models were adjusted for age, sex, race/ethnicity, education, family income, body mass index, smoking status, and alcohol consumption.

## Data Availability

The data presented in this study are openly available on the CDC NHANES site at https://wwwn.cdc.gov/nchs/nhanes/continuousnhanes/overview.aspx?BeginYear=2017 (accessed on 8 June 2026).

## Supplementary Materials

The following supporting information can be downloaded at: https://www.mdpi.com/article/10.3390/jox16040128/s1, Figure S1: Trace plots of kernel variance (σ^2^) parameters from the survey-weighted Bayesian Kernel Machine Regression (BKMR) model across four independent Markov chain Monte Carlo (MCMC) chains (20,000 iterations per chain). Each panel rep-resents one exposure-specific kernel variance parameter. Persistent separation of the chains and the absence of stable overlap indicate poor chain mixing and failure to achieve satisfactory posterior convergence; Figure S2: Unadjusted prevalence of self-reported diabetes across blood lead exposure quartiles stratified by race/ethnicity in the measured-only analytic sample (NHANES 2017–2018, N = 1648). Bars represent the observed prevalence of self-reported diabetes within each blood lead quartile for individual racial/ethnic groups. Estimates are presented for de-scriptive purposes only and were not adjusted for covariates or the complex NHANES survey design; Figure S3: Sex-stratified survey-weighted logistic regression analyses evaluating associations between standardized blood concen-trations of lead, cadmium, PFOA, PFOS, and mercury and prevalent self-reported diabetes among male (N = 836) and female (N = 862) participants. Odds ratios (ORs) and 95% confidence intervals are shown per one-standard-deviation increase in log-transformed exposure concentrations. Models were adjusted for age, race/ethnicity, educational attain-ment, income-to-poverty ratio, body mass index, smoking status, and alcohol consumption and incorporated the NHANES complex survey design (WTSB2YR, SDMVPSU, SDMVSTRA). Estimates should be interpreted cautiously be-cause subgroup analyses had reduced statistical power; Figure S4: Race/ethnicity-stratified survey-weighted logistic regression evaluating associations between environmental exposures and prevalent self-reported diabetes among Non-Hispanic White (N = 591), Non-Hispanic Black (N = 361), and Non-Hispanic Asian (N = 216) participants. Odds ratios (ORs) and 95% confidence intervals are shown per one standard deviation increase in each log-transformed, standardized exposure, adjusted for age, sex, educational attainment, in-come-to-poverty ratio, body mass index, smoking status, and alcohol consumption. Survey design incorporated PFAS subsample weights (WTSB2YR), primary sampling unit clustering (SDMVPSU), and stratification (SDMVSTRA). Esti-mates should be interpreted with caution given small within-stratum sample sizes and reduced statistical power. PFOA: perfluorooctanoic acid; PFOS: perfluorooctanesulfonic acid; Figure S5: Gelman–Rubin convergence diagnostics (potential scale reduction factor, R-hat) for naïve and survey-weighted Bayesi-an Kernel Machine Regression (BKMR) models. R-hat values were calculated from four independent Markov chain Mon-te Carlo (MCMC) chains (20,000 iterations per chain). Values approaching 1.0 indicate satisfactory convergence, where-as larger values indicate failure of convergence. The dashed vertical line denotes the conventional convergence threshold (R-hat = 1.10); Figure S6: Gelman–Rubin convergence diagnostics (R-hat point estimates and upper 97.5% confidence limits) for naïve and de-sign-aware Bayesian kernel machine regression (BKMR) models fitted to the measured-only NHANES 2017–2018 ana-lytic sample (N = 1648). Four independent Markov chain Monte Carlo (MCMC) chains were run for 20,000 iterations each. Orange dashed vertical lines indicate the commonly used convergence thresholds (R-hat = 1.10 for point estimates and upper 97.5% confidence limit = 1.20). Values below these thresholds indicate satisfactory convergence; Figure S7: Outlier sensitivity analysis comparing survey-weighted logistic regression results from the full analytic sample (N = 1648, blue circles) and a trimmed sample with participants above the 99th percentile of each exposure distribution ex-cluded (N = 1572, red triangles). Odds ratios (ORs) and 95% confidence intervals are shown per one standard deviation increase in each log-transformed, standardized exposure, adjusted for age, sex, race/ethnicity, educational attainment, income-to-poverty ratio, BMI, smoking status, and alcohol consumption. Outliers were defined as observations exceed-ing the 99th percentile of the within-sample exposure distribution for each exposure simultaneously. The consistency of effect estimates across both samples confirms that findings were not materially influenced by extreme exposure values. Survey design incorporated PFAS subsample weights (WTSB2YR), primary sampling unit clustering (SDMVPSU), and stratification (SDMVSTRA); Table S1: Comparison of complete-case and multiple imputation analyses for the survey-weighted logistic regression model (NHANES 2017–2018); Table S2: WQS regression exposure weights and BKMR posterior inclusion probabilities (PIP) across four independent MCMC chains (NHANES 2017–2018, N = 1648); Table S3: Multicollinearity assessment and BKMR convergence diagnostics (NHANES 2017–2018, N = 1648).

## Abbreviations

BKMR: Bayesian Kernel Machine Regression; WQS: Weighted Quantile Sum; qgcomp: quantile g-computation; PFAS: Per- and Polyfluoroalkyl Substances; PFOA: Perfluorooctanoic Acid; PFOS: Perfluorooctane Sulfonate; NHANES: National Health and Nutrition Examination Survey; PIP: Posterior Inclusion Probability; MICE: Multiple Imputation by Chained Equations; PMM: Predictive Mean Matching; MAR: Missing at Random; AUC: Area Under the Curve; AIC: Akaike Information Criterion; ROC: Receiver Operating Characteristic; RR: Risk Ratio; OR: Odds Ratio; BMI: Body Mass Index; CDC: Centers for Disease Control and Prevention; NCHS: National Center for Health Statistics; MCMC: Markov Chain Monte Carlo; SD: Standard Deviation; CI: Confidence Interval; PCA: Principal Component Analysis; MLR: Multiple Linear Regression; BLL: Blood Lead Level.
