# Supplementary material for: Combined Effects of Metals and PFAS Exposure on Prevalent Diabetes
Source: J Xenobiot. 2026 Jul 10;16(4):128. doi: 10.3390/jox16040128 (PMC13398037; doi:10.3390/jox16040128)
Supplement: Supplementary file 1 [file jox-16-00128-s001.zip › jox-4346221-supplementary.pdf]

Supplementary Materials: Combined Effects of Metals and PFAS Exposure on Prevalent Diabetes

Rifa Tasnia and Emmanuel Obeng-Gyasi

Supplementary Tables:

The primary survey-weighted logistic regression model was re-estimated using only participants with complete data on all covariates (complete-case analysis, N = 1,274) to determine whether results were sensitive to the multiple imputation technique used to address missing covariate data. The complete-case and multiple-imputation analyses showed a high degree of consistency in the direction and approximate magnitude of all five exposure-diabetes relationships. Under both methods, blood lead and blood cadmium continued to be strongly and negatively correlated with diabetes. The key conclusions were not significantly affected by missing covariate data, as confirmed by the comparability of point estimates and confidence intervals.

Table S1. Comparison of complete-case and multiple imputation analyses for the survey-weighted logistic regression model (NHANES 2017–2018).

Exposure	Complete-case OR (95% CI)	*p*-value	Imputed OR (95% CI)	*p*-value
PFOA	0.66 (0.42–1.05)	0.089	0.66 (0.42–1.04)	0.092
PFOS	1.04 (0.85–1.28)	0.695	1.05 (0.86–1.29)	0.631
**Lead**	**0.26 (0.08–0.79)**	**0.033**	**0.25 (0.09–0.71)**	**0.020**
**Cadmium**	**0.72 (0.53–0.98)**	**0.041**	**0.71 (0.54–0.94)**	**0.028**
Mercury	0.97 (0.73–1.29)	0.831	0.96 (0.73–1.26)	0.751

Table S2 presents WQS exposure-specific weights from the dual-index model alongside BKMR posterior inclusion probabilities (PIPs) across four independent chains of 20,000 MCMC iterations. Comparing these two complementary methods reveals a generally consistent pattern of variable importance across analytical frameworks.

Lead dominated the negative WQS index (weight = 0.458, 45.8% of total) and showed a near-certain BKMR PIP of 1.000 across all four chains with zero variation. This indicates that lead's dominant role in the mixture is confirmed by both approaches. PFOS dominated the positive WQS index (weight = 0.354, 35.4%) and showed a near-certain BKMR PIP of 0.997, despite its non-significant individual logistic regression OR (1.05, p = 0.631). The difference between the mixture-based and logistic regression results for PFOS highlights the usefulness of BKMR and WQS in discovering mixture-level contributions that marginal regression is unable to detect.

PFOA, cadmium, and mercury showed lower and more variable PIPs across BKMR chains, and moderate WQS weights in both indices, suggesting comparatively weaker and less certain contributions to the joint exposure–response surface. The cross-method consistency in identifying lead and PFOS as the most influential mixture components substantially strengthens confidence in the variable importance findings of this study.

Table S2. WQS regression exposure weights and BKMR posterior inclusion probabilities (PIP) across four independent MCMC chains (NHANES 2017–2018, N = 1,648).

Exposure	Negative WQS Weight	Positive WQS Weight	Mean PIP	SD
**Lead**	**0.458**	0.064	**1.000**	0.000
**PFOS**	0.096	**0.354**	**0.997**	0.003

Exposure	Negative WQS Weight	Positive WQS Weight	Mean PIP	SD
PFOA	0.128	0.231	0.469	0.115
Cadmium	0.136	0.187	0.340	0.115
Mercury	0.163	0.168	0.337	0.094
**Sum**	**1.000**	**1.000**	—	—

Bold values indicate the dominant contributor within each WQS index (weight > equal-weight threshold of 0.20) or high BKMR inclusion probability (PIP ≥ 0.99). Negative WQS index: OR = 0.45 (95% CI: 0.23–0.88), $p < 0.001$. Positive WQS index: OR = 0.90 (95% CI: 0.47–1.71), $p = 0.534$. WQS conducted without survey weights (secondary sample-level analysis). Mean PIP and SD represent the mean and standard deviation of PIPs across four independent MCMC chains of 20,000 iterations each (naive BKMR). WQS: weighted quantile sum; BKMR: Bayesian kernel machine regression; PIP: posterior inclusion probability; SD: standard deviation; PFOA: perfluorooctanoic acid; PFOS: perfluorooctanesulfonic acid; OR: odds ratio; CI: confidence interval.

Table S3 presents two sets of methodological diagnostics for the five environmental exposures: variance inflation factors (VIF) assessing multicollinearity, and Gelman–Rubin R-hat statistics assessing BKMR convergence.

For multicollinearity, all VIF values were below 1.21, confirming the absence of meaningful multicollinearity among the five exposure variables. This finding is consistent with the Spearman rank correlation analysis (Figure 3 in main text), which showed that pairwise correlations among exposures were generally weak to moderate ($\varrho \leq 0.63$), and supports the validity of including all five exposures simultaneously in the logistic regression and mixture models without bias.

For BKMR convergence, the naive model showed R-hat values between 1.03 and 1.08 for all five exposures, all below the conventional threshold of 1.10. This confirms satisfactory convergence. Lead showed the highest naive R-hat point estimate (1.08) with an upper 97.5% confidence limit of 1.22, which is considered borderline but acceptable. The design-aware BKMR model, in contrast, showed severe non-convergence across all five exposures, with R-hat values ranging from 3.05 (mercury) to 13.16 (cadmium). The upper 97.5% confidence limits for the design-aware model were extremely wide — reaching 70.78 for cadmium and 56.63 for lead — reflecting highly unstable posterior estimation. These findings confirm that the naive BKMR achieved reliable convergence and support its use for primary inference, while the design-aware BKMR results are presented for methodological transparency only.

Table S3. Multicollinearity assessment and BKMR convergence diagnostics (NHANES 2017–2018, N = 1,648).

Exposure	VIF	Interpretation	Naïve R-hat	Naïve Upper CI	Design-aware R-hat	Design-aware Upper CI
Lead	1.03	No multicollinearity	**1.08**	1.22	11.70	56.63
PFOS	1.18	No multicollinearity	**1.03**	1.07	7.48	17.51
PFOA	1.18	No multicollinearity	**1.07**	1.14	7.90	45.38
Cadmium	1.03	No multicollinearity	**1.05**	1.12	**13.16**	70.78
Mercury	1.16	No multicollinearity	**1.06**	1.12	3.05	7.14

VIF calculated from ordinary least squares regression of each exposure on all remaining exposures; values < 2.0 indicate no meaningful multicollinearity. Naive R-hat: Gelman–Rubin potential scale reduction factor from naive (unweighted) BKMR across four independent chains of 20,000 iterations each; values < 1.10 indicate convergence (conventional threshold). Design-aware R-hat: from survey- weighted BKMR; all values substantially exceeded the convergence threshold, confirming non-convergence. Upper CI: upper 97.5% confidence limit of the R-hat statistic. Bold naive R-hat values indicate converged chains (< 1.10); bold design-aware R-hat indicates the most severe non-convergence. VIF: variance inflation factor; BKMR: Bayesian kernel machine regression; PFOA: perfluorooctanoic acid; PFOS: perfluorooctanesulfonic acid.

Supplementary Figures:

Convergence of the survey-weighted BKMR model was evaluated using trace plots from four independent MCMC chains (Supplementary Figure S2). Rather than converging toward a common stationary distribution, the chains remained clearly separated throughout the sampling process and exhibited substantial fluctuations across iterations. Persistent divergence was observed for all exposure components, particularly cadmium and lead, indicating poor chain mixing and unstable posterior estimation. These findings demonstrate that the survey-weighted BKMR model failed to achieve satisfactory convergence despite 20,000 iterations per chain. Consequently, the survey-weighted BKMR results were retained only as a methodological sensitivity analysis and were not used for substantive interpretation.

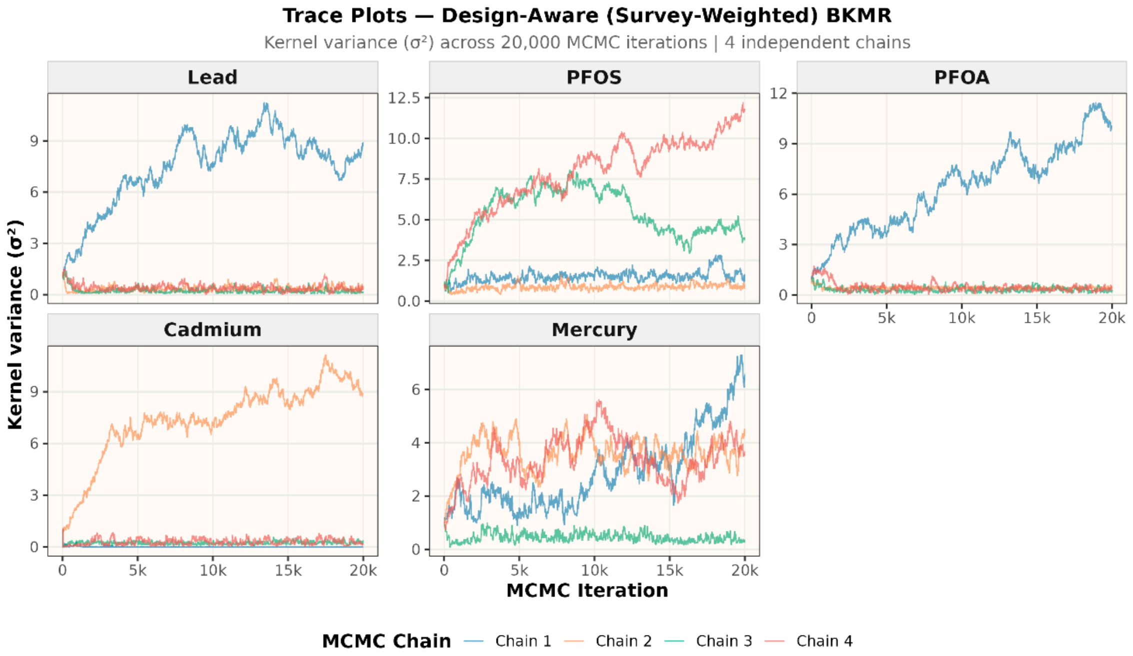

Figure S1. Trace plots of kernel variance (σ²) parameters from the survey-weighted Bayesian Kernel Machine Regression (BKMR) model across four independent Markov chain Monte Carlo (MCMC) chains (20,000 iterations per chain). Each panel represents one exposure-specific kernel variance parameter. Persistent separation of the chains and the absence of stable overlap indicate poor chain mixing and failure to achieve satisfactory posterior convergence.

Figure S2 presents the crude prevalence of self-reported diabetes across blood lead exposure quartiles within individual racial/ethnic groups. Differences in prevalence were observed between racial/ethnic groups and across exposure quartiles; however, no consistent monotonic pattern was evident across all subgroups. Because these estimates are unadjusted and several subgroup-specific sample sizes were relatively small, they should be interpreted as descriptive rather than inferential. Formal evaluation of exposure–diabetes associations was performed using the survey-weighted multivariable logistic regression models presented in the main manuscript, which appropriately accounted for the complex sampling design and potential confounding.

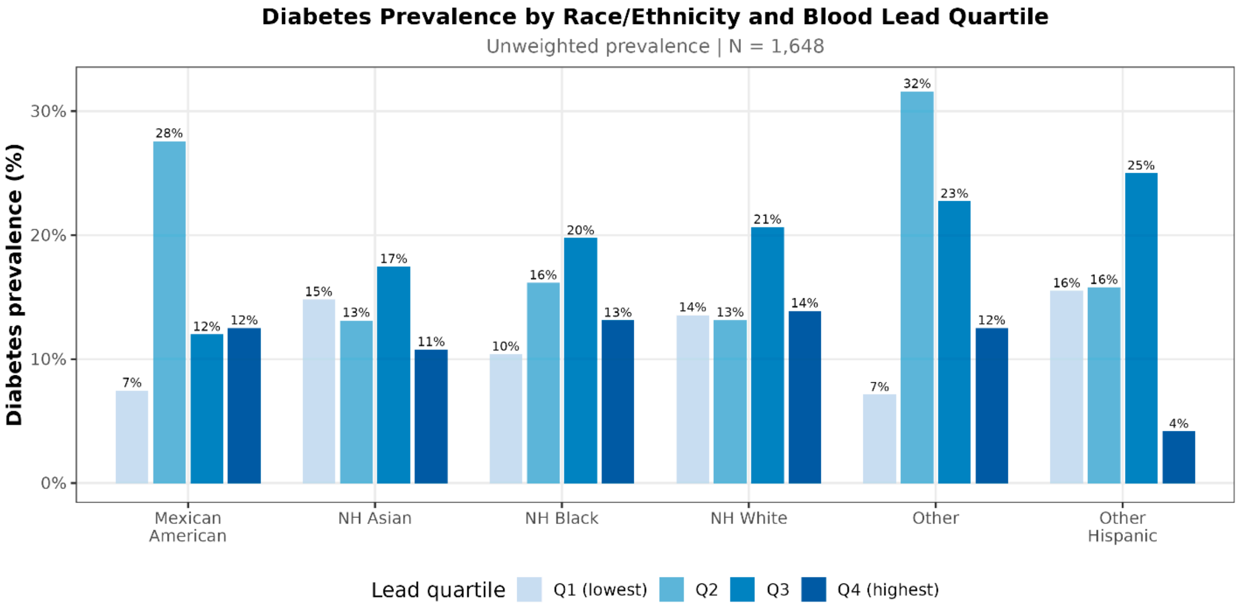

Figure S2. Unadjusted prevalence of self-reported diabetes across blood lead exposure quartiles stratified by race/ethnicity in the measured-only analytic sample (NHANES 2017–2018, N = 1,648). Bars represent the observed prevalence of self-reported diabetes within each blood lead quartile for individual racial/ethnic groups. Estimates are presented for descriptive purposes only and were not adjusted for covariates or the complex NHANES survey design. .

Sex-stratified survey-weighted logistic regression results are presented in Supplementary Figure S3. The direction of associations was broadly consistent between males and females across all five exposures. Lead and cadmium demonstrated inverse associations in both sexes, with point estimates below the null in males (Lead OR = 0.37; Cadmium OR = 0.28) and females (Lead OR = 0.30; Cadmium OR = 0.40), though neither reached statistical significance within strata, likely reflecting reduced statistical power compared to the combined analysis. PFOA showed a non-significant inverse trend in both sexes. PFOS and mercury were near the null in both males and females. No substantial evidence of sex-specific effect modification was observed, and the overall pattern of associations was consistent with the primary analysis.

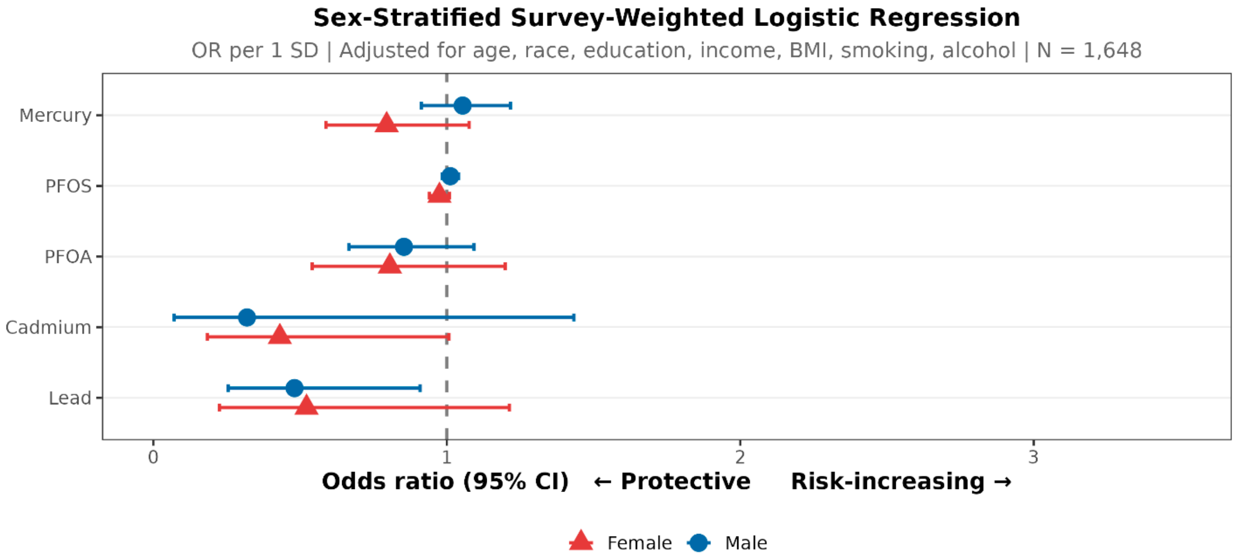

Figure S3. Sex-stratified survey-weighted logistic regression analyses evaluating associations between standardized blood concentrations of lead, cadmium, PFOA, PFOS, and mercury and prevalent self-reported diabetes among male (N = 836) and female (N = 862) participants. Odds ratios (ORs) and 95% confidence intervals are shown per one-standard-deviation increase in log-transformed exposure concentrations. Models were adjusted for age, race/ethnicity, educational attainment, income-to-poverty ratio, body mass index, smoking status, and alcohol consumption and incorporated the NHANES complex survey design (WTSB2YR, SDMVPSU, SDMVSTRA). Estimates should be interpreted cautiously because subgroup analyses had reduced statistical power.

Race/ethnicity-stratified survey-weighted logistic regression results are presented in Supplementary Figure S4. Among Non-Hispanic White participants, lead demonstrated the strongest inverse association (OR = 0.19, 95% CI: 0.07–0.52), consistent with the overall primary analysis finding. Among Non-Hispanic Black participants, lead also showed an inverse association (OR = 0.38), though with wider confidence intervals reflecting the smaller subgroup sample size. Among Non-Hispanic Asian participants, cadmium showed an unexpectedly positive association (OR ≈ 2.1); however, this estimate should be interpreted with considerable caution given the small subgroup size (N = 216), sparse event counts, and wide confidence interval spanning nearly the full range of the x-axis. PFOS and mercury were near the null across all three racial/ethnic groups. The overall pattern of inverse associations for lead was directionally consistent across racial/ethnic subgroups, supporting the robustness of the primary finding, though formal tests of interaction by race/ethnicity were not conducted given insufficient statistical power within strata.

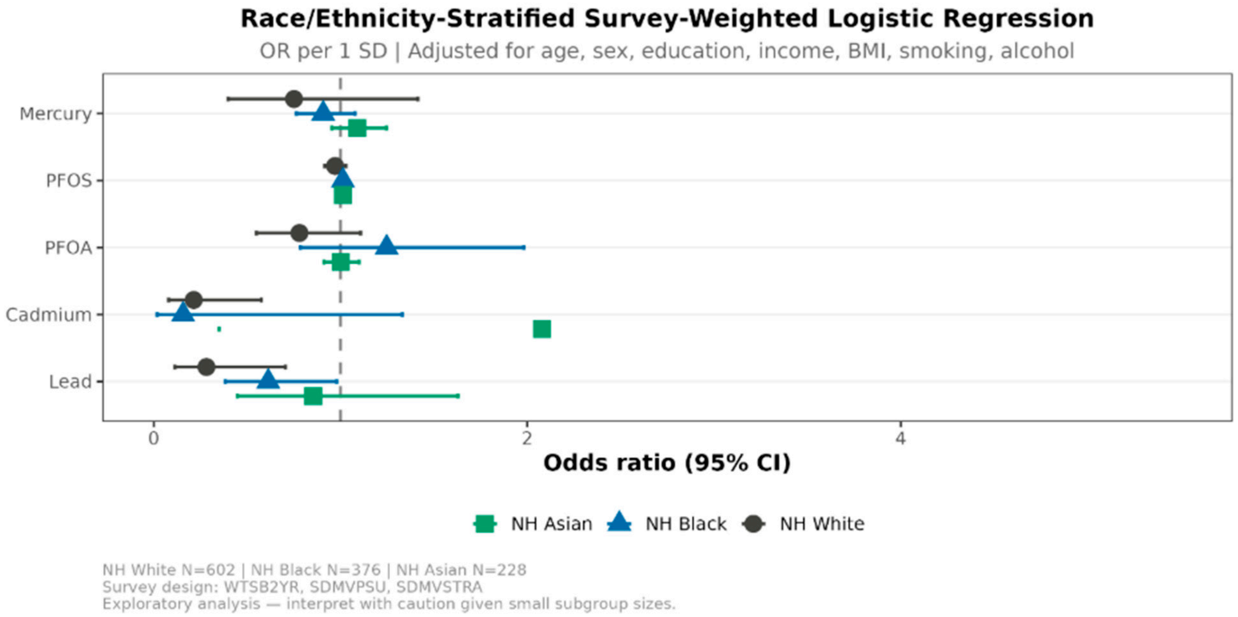

Figure S4. Race/ethnicity-stratified survey-weighted logistic regression evaluating associations between environmental exposures and prevalent self-reported diabetes among Non-Hispanic White (N = 591), Non-Hispanic Black (N = 361), and Non-Hispanic Asian (N = 216) participants. Odds ratios (ORs) and 95% confidence intervals are shown per one standard deviation increase in each log-transformed, standardized exposure, adjusted for age, sex, educational attainment, income-to-poverty ratio, body mass index, smoking status, and alcohol consumption. Survey design incorporated PFAS subsample weights (WTSB2YR), primary sampling unit clustering (SDMVPSU), and stratification (SDMVSTRA). Estimates should be interpreted with caution given small within-stratum sample sizes and reduced statistical power. PFOA: perfluorooctanoic acid; PFOS: perfluorooctanesulfonic acid.

The naïve BKMR model demonstrated satisfactory convergence for all five exposures, with R-hat values ranging from 1.03 to 1.08, indicating good agreement among the four independent Markov chain Monte Carlo (MCMC) chains and stable posterior estimation. In contrast, the survey-weighted BKMR model exhibited markedly elevated R-hat values (3.05–13.16) across all exposure-specific kernel variance parameters, substantially exceeding the accepted convergence threshold of 1.10. These findings are consistent with the corresponding trace plots (Supplementary Figure S1), which showed poor chain mixing and lack of stationarity. Collectively, the convergence diagnostics indicate that the survey-weighted BKMR model failed to adequately sample the posterior distribution, precluding reliable statistical inference. Consequently, the survey-weighted BKMR analysis was retained only as a methodological sensitivity analysis, whereas interpretation of nonlinear mixture effects and variable importance was based on the converged naïve BKMR model.

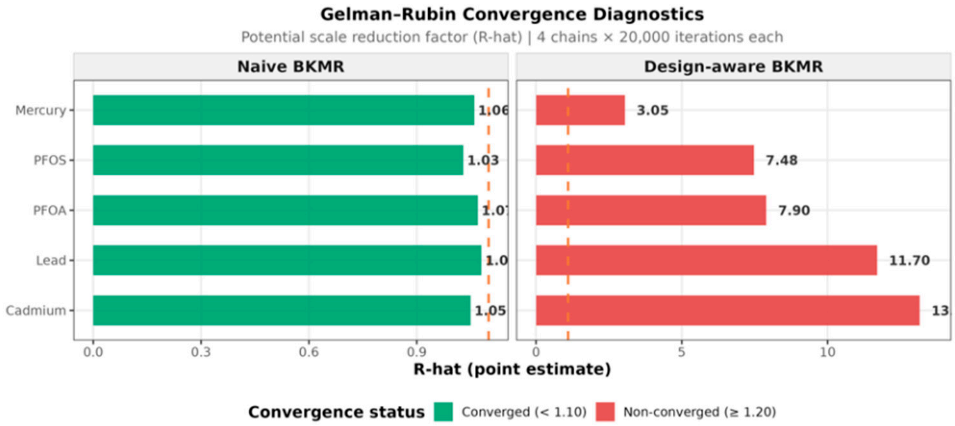

Figure S5. Gelman–Rubin convergence diagnostics (potential scale reduction factor, R-hat) for naïve and survey-weighted Bayesian Kernel Machine Regression (BKMR) models. R-hat values were calculated from four independent Markov chain Monte Carlo (MCMC) chains (20,000 iterations per chain). Values approaching 1.0 indicate satisfactory convergence, whereas larger values indicate failure of convergence. The dashed vertical line denotes the conventional convergence threshold (R-hat = 1.10).

The Gelman–Rubin convergence diagnostics demonstrated satisfactory convergence for all parameters in the naïve BKMR model, with R-hat point estimates below 1.10 and upper 97.5% confidence limits remaining below the recommended threshold of 1.20 (Figure 6). In contrast, the design-aware BKMR model exhibited substantially elevated R-hat values and upper confidence limits for all exposure parameters, indicating poor mixing of the Markov chains and lack of convergence despite extended MCMC sampling. Because reliable Bayesian inference requires adequate convergence across independent chains, inference from BKMR was based on the converged naïve model, whereas the primary epidemiologic conclusions were derived from the survey-weighted logistic regression analyses.

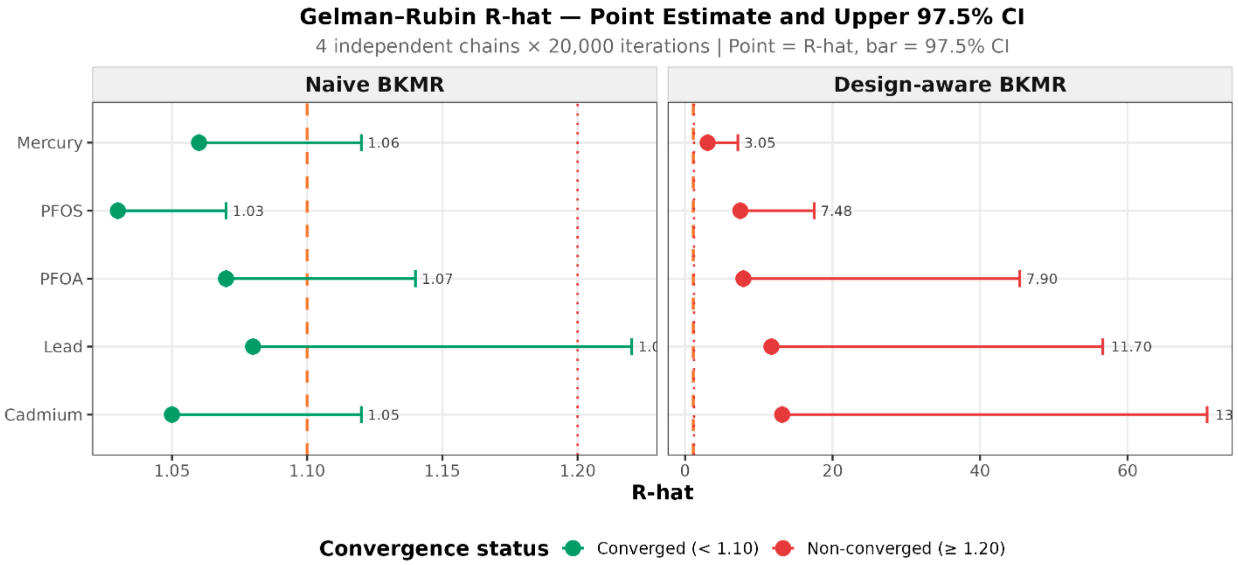

Figure S6. Gelman–Rubin convergence diagnostics (R-hat point estimates and upper 97.5% confidence limits) for naïve and design-aware Bayesian kernel machine regression (BKMR) models fitted to the measured-only NHANES 2017–2018 analytic sample (N = 1,648). Four independent Markov chain Monte Carlo (MCMC) chains were run for 20,000 iterations each. Orange dashed vertical lines indicate the commonly used convergence thresholds (R-hat = 1.10 for point estimates and upper 97.5% confidence limit = 1.20). Values below these thresholds indicate satisfactory convergence.

To assess whether primary findings were sensitive to high-exposure outliers, the survey-weighted logistic regression model was re-estimated after excluding participants above the 99th percentile of each exposure distribution simultaneously, resulting in a trimmed analytic sample of N = 1,572 (Supplementary Figure S7). Blood lead and blood cadmium remained significantly and inversely associated with self-reported diabetes in the trimmed sample, with effect estimates consistent in direction and approximate magnitude with those from the full analytic sample. PFOA, PFOS, and mercury remained nonsignificant in both the full and trimmed samples. The consistency of findings across analytic samples confirms that the primary results were not materially influenced by extreme exposure values at the upper tail of the distribution.

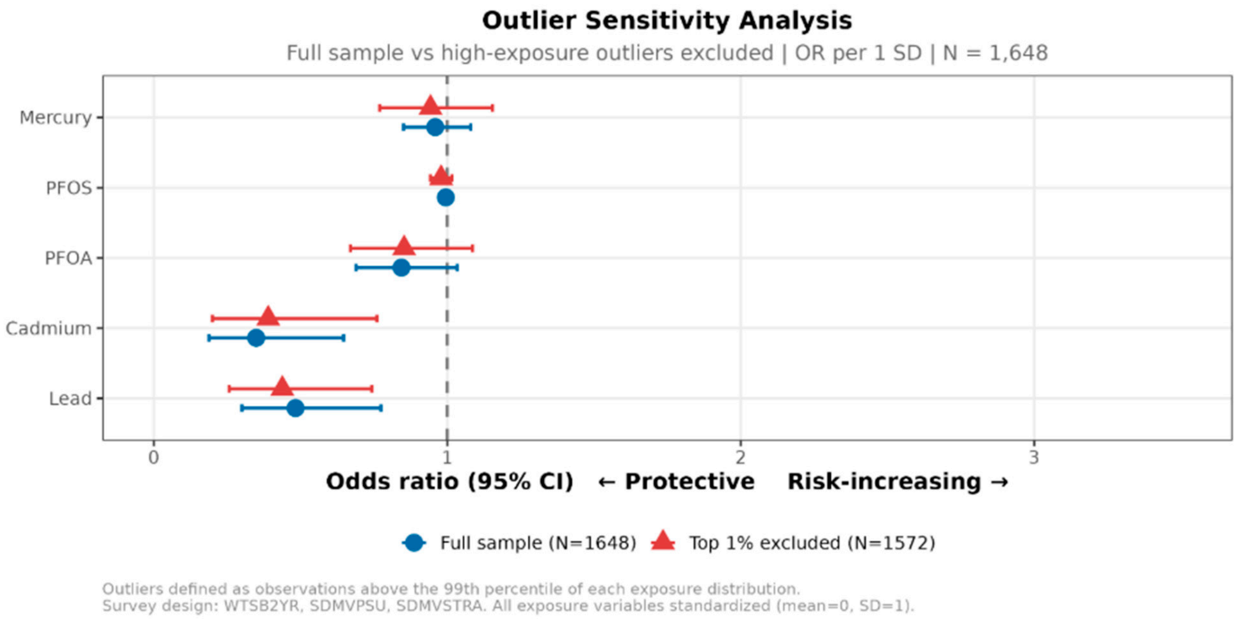

Figure S7. Outlier sensitivity analysis comparing survey-weighted logistic regression results from the full analytic sample (N = 1,648, blue circles) and a trimmed sample with participants above the 99th percentile of each exposure distribution excluded (N = 1,572, red triangles). Odds ratios (ORs) and 95% confidence intervals are shown per one standard deviation increase in each log-transformed, standardized exposure, adjusted for age, sex, race/ethnicity, educational attainment, income-to-poverty ratio, BMI, smoking status, and alcohol consumption. Outliers were defined as observations exceeding the 99th percentile of the within-sample exposure distribution for each exposure simultaneously. The consistency of effect estimates across both samples confirms that findings were not materially influenced by extreme exposure values. Survey design incorporated PFAS subsample weights (WTSB2YR), primary sampling unit clustering (SDMVPSU), and stratification (SDMVSTRA).
